# Metal-based molecules in the treatment of cancer: From bench to bedside

**DOI:** 10.32604/or.2024.057019

**Published:** 2025-03-19

**Authors:** GIULIANO BERNAL, GISELA AQUEA, SEBASTIÁN RAMÍREZ-RIVERA

**Affiliations:** Laboratory of Molecular and Cellular Biology of Cancer, Department of Biomedical Sciences, Faculty of Medicine, Universidad Católica del Norte, Coquimbo, 1781421, Chile

**Keywords:** Metal-based drugs, Cancer treatment, Chemotherapy

## Abstract

Cancer remains one of the leading causes of death in the world, with more than 9 million deaths in 2022, a number that continues to rise. This highlights the urgent need for the development of new drugs, with enhanced antitumor capabilities and fewer side effects. Metal-based drugs have been used in clinical practice since the late 1970s, beginning with the introduction of cisplatin. Later, two additional platinum-based molecules, carboplatin, and oxaliplatin, were introduced, and all three continue to be widely used in the treatment of various cancers. However, despite their significant anticancer activity, the undesirable side effects of these drugs have motivated the scientific community to explore other metal-based complexes with greater anticancer potential and fewer adverse effects. In this context, metals such as ruthenium, copper, gold, zinc, palladium, or iridium, present promising alternatives for the development of new anticancer agents. Unfortunately, although thousands of metal-based drugs have been synthesized and tested both *in vitro* and in animal models, only a few ruthenium-based drugs have entered clinical trials in recent years. Meanwhile, many other molecules with comparable or even greater anticancer potential have not advanced beyond the laboratory stage. In this review, we will revisit the mechanisms of action and anticancer activities of established platinum-based drugs and explore their use in recent clinical trials. Additionally, we will examine the development of potential new metal-based drugs that could one day contribute to cancer treatment worldwide.

## Introduction

Cancer is one of the most significant non-communicable diseases (NCDs) globally, with nearly 20 million new cases and 9.7 million deaths in 2022. By 2050, a projected increase of 77% is expected, leading to over 35 million new cancer cases annually [1]. In light of this alarming trend, there is a critical need for the development of new drugs that can effectively combat the rising incidence of cancer, offering enhanced antitumor capabilities and fewer side effects.

The development of metal-based drugs in cancer therapy dates back to the mid-1960s when Barnett Rosenberg discovered the antiproliferative activity of cisplatin [2,3]. Although the molecule was discovered in 1845 by Michele Peyrone, it was not until 1978 that the Food and Drug Administration (FDA) approved cisplatin for use in cancer therapy [4]. Later, additional platinum drugs, such as carboplatin and oxaliplatin, were introduced [2,4]. These platinum drugs have been extensively used to treat different types of cancer worldwide. However, resistance to these drugs has been observed in many cases, necessitating to combine it with other drugs, such as tyrosine kinase inhibitors (TKIs) (imatinib, nilotinib) [5], taxanes (paclitaxel) [6], ribonucleotide reductase inhibitors (Gemcitabine) [7], phosphatidylinositol 3-kinase (PI3K) inhibitors (Alpelisib) [8], among others. These combinations not only improve the anticancer activity of platinum drugs but also reduce the side effects experienced by patients.

Studies have also demonstrated that synergistic use of platinum drugs with natural compounds, such as oxyresveratrol [9], isovitexin [10], limonene [11], or extracts from fermented ginger [12], *Vernonia calvoana* [13], *Platycodon grandiflorus* [14] phenylethanol glycosides from Herba Cistanche [15], or the essential oil of Ylang ylang *(Cananga odorata)* [16], can enhance efficacy and reduce toxicity.

In a recent article, genes potentially linked to resistance to platin drugs in ovarian cancer were investigated in 90 chemoresistant and 197 chemosensitive tissues. The findings, correlated with data of 1347 patients, revealed that a high expression of PARD6B and STAT5A or a low expression of SOS1, MSH6, and STAT5A could serve as reliable markers for predicting resistance to platinum-based drugs [17].

Since the development of cisplatin, carboplatin, and oxaliplatin, a wide array of new platinum-based drugs has been designed using “structure-activity relationship” models. However, only a few have advanced to clinical trials [2,18]. With the global rise in cancer incidence, other metal-based complexes, incorporating ions such as ruthenium, copper, gold, zinc, palladium, and iridium, have attracted significant interest as potential anticancer agents [18–22].

In this review, we will explore the structures and potential mechanism of action of metal-based drugs, including those containing platinum, ruthenium, copper, gold, palladium, zinc, and iridium, as well as their potential use in cancer treatment.

## Biochemical and Clinical Properties of Metal-Based Drugs

Metal-based complexes exhibit remarkable chemical diversity and versatility, which depend on factors such as the coordinated metal, its oxidation state, the number and type of ligands, and specific magnetic and/or optical properties [3]. Metals like platinum, ruthenium, copper, gold, palladium, zinc, or iridium possess notable characteristics, including redox activity, variable coordination modes, and reactivity towards organic substrates, principally proteins or DNA. These properties grant metal complexes significant therapeutic potential in cancer treatment [18].

### Platinum compounds

Cisplatin (Table 1), the first metal-based drug used in the treatment of cancer, exerts its antiproliferative effects primarily by binding to DNA, blocking DNA replication and transcription, and triggering caspase activation and apoptosis [2,4,23]. In the bloodstream, cisplatin is primarily bound to plasma proteins, such as albumin, and is subsequently transported across the plasma membrane by transporters such as copper transporter-1 and -2 (Ctr1, Ctr2), the P-type copper-transporting ATPases ATP7A and ATP7B, and the organic cation transporter-2 (OCT2) [24–27]. Once inside the cell, cisplatin undergoes monoaquation, where one of the chloride ligands is replaced by a water molecule due to the lower chloride concentration in the cytoplasm (4–20 mM) compared to the blood (approximately 100 mM) [2,28]. The monoaquated platinum acts as a potent electrophile, reacting with nitrogen donor atoms in DNA, forming intra- and inter-strand crosslinks that induce DNA adducts and damage [29,30]. This damage activates repair mechanisms, including mismatch repair proteins and high-mobility group box (HMGB) proteins [24,25]. When the damage surpasses the cell’s repair capacity, apoptotic pathways are activated, involving the expression of p53 and Bcl2 Associated X Protein (BAX, part of the Bcl-2 family proteins), the cytochrome c release, and the activation of caspase-3, -7 and -9, leading to apoptosis [2,19,24,30,31].

**Table 1:** The table represents some examples of the anti-cancer activity of platinum-based compounds

Structure	Platinum compounds	*In vitro* or *in vivo* activity	Mechanism of action	Reference
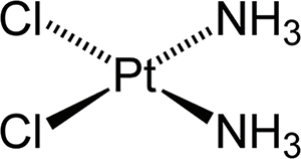	Cisplatin	It is used to treat various types of cancer, including breast, ovarian, testicular, head and neck, esophageal, lung, bladder, and brain	It enters cells through transporters Ctr1, Ctr2, ATP7A, ATP7B, and OCT2. It binds to DNA, and induces apoptosis through P53, BAX, and caspase-3, -7, and -9	[2,19,24,30,31]
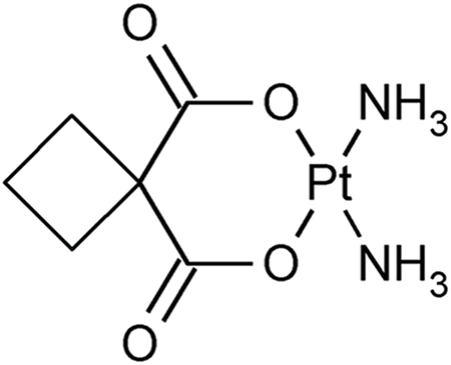	Carboplatin	It is the treatment of choice for TNBC. It is used to treat ovarian, lung, and certain head-and-neck cancers	It enters cells through the transporters Crt1, ATP7A, and ATP7B. It can induce the expression of miR-145 and then p53, along with binding to DNA, causing deformation and initiating apoptosis	[60]
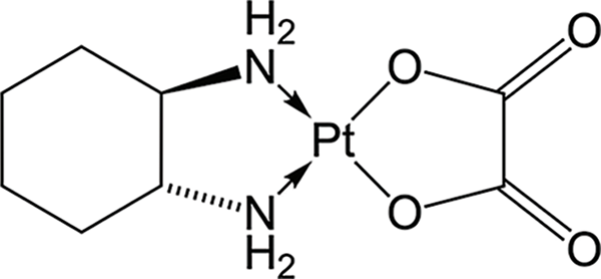	Oxaliplatin	Is the treatment of election for colorectal cancer	It enters cells via the CTR1 transporter. Oxaliplatin inhibits RNA Pol I, leading to rRNA silencing and nucleolar disruption, likely mediated by the DNA damage response kinases ATM and ATR	[60]
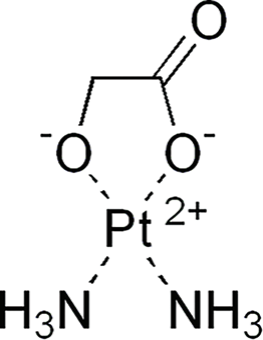	Nedaplatin	It has anticancer activity against cervical, small- and non-small cell lung, breast, ovarian, and testicular cancers,	Nedaplatin has the same therapeutic mechanisms as cisplatin but is more water-soluble than cisplatin	[73]
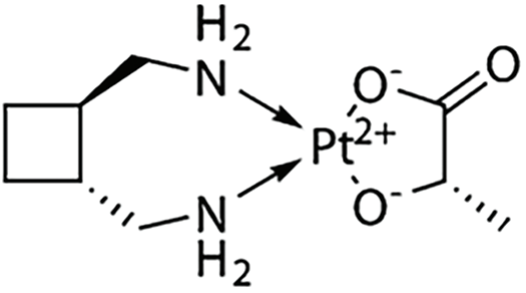	Lobaplatin	Tested in bladder cancer cell lines T24 and 5637	It induces apoptosis by the regulation of Bcl-2 and BAX expression and inhibition of the PI3K/Akt signaling pathway	[75]
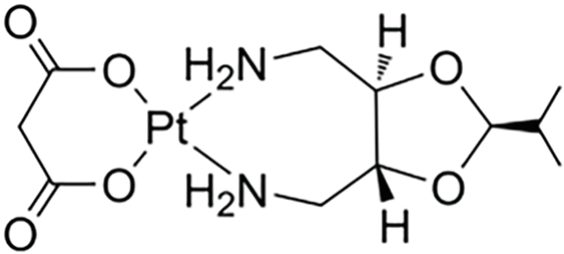	Heptaplatin	Tested in colorectal cancer cell HCT116 and HT2	Induces an inhibitory response in the G1 phase of the cell cycle in these cells	[76,77]
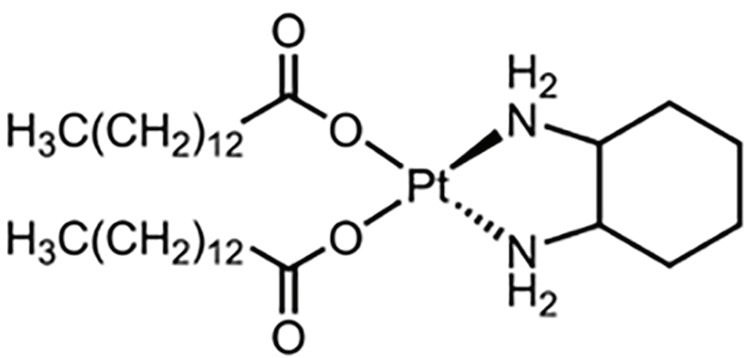	Miriplatin	It was synthesized for the treatment of unresectable hepatocellular carcinoma. It has also tested in human pancreatic cancer cell lines AsPC-1, BxPC-3, MIA-PaCa-2, PANC-1, and SU.86.86	It inhibits POLG and TFAM-mediated mtDNA replication	[78]

Cisplatin is used to treat various cancers, including breast, ovarian, testicular, head and neck, esophageal, lung, bladder, and brain cancers [2]. However, cisplatin is associated with severe adverse effects, particularly nephrotoxicity and ototoxicity [32]. Cisplatin-induced nephrotoxicity occurs in approximately 28%–36% of patients due to drug accumulation in proximal tubular cells, which impairs the expression of glucose, amino acid, magnesium, and water transporters, reduces renal blood flow, and induces acute kidney injury (AKI) [33,34]. Nephrotoxicity is more frequent in children than in adults. A recent study involving 159 children treated with cisplatin revealed that 46% developed AKI [35]. Nonetheless, Several natural compounds have demonstrated cytoprotective effects against AKI, including curcumin (a Curcuma derivative) [36]; Gastrodin (the main active ingredient of *Gastrodia elata* Blume) [37]; ginsenosides (the main active components of ginseng roots) [38]; asiatic acid (triterpenoid extracted from *Centella asiatica*) [39]; betulinic acid (a triterpene isolated from *Silene succulenta* Forssk) [40]; Oleanolic acid acetate (a triterpene isolated from *Vigna angularis*) [41], isorhamnetin (flavanol derived from *Hippophae rhamnoides* L. and *Ginkgo biloba* L.) [42]; Kaempferide (flavanol found in the rhizome of *Kaempferia galanga, Chromolaena odorata*, *Alpinia conchigera*, and *Hippophae rhamnoides* L.) [43]; and isoliquiritigenin (flavonoid extracted from *Glycyrrhiza glabra*, *Allium ascalonicum*, and *Glycine max*) [44]. Similar nephroprotective effects have been observed with extracts from *Achyranthes aspera* root [45]; *Olea europaea* [46]; *Clinacanthus nutans* [47]; and *Primula vulgaris* leaves [48].

In these cases, the protective mechanisms involve a reduction in serum levels of blood urea nitrogen (BUN) and creatinine, along with an increase in glutathione (GSH) levels and activation of Nuclear factor erythroid 2-related factor 2 (Nrf2), a key regulator of cellular redox homeostasis. Furthermore, these compounds reduce serum proinflammatory cytokines, including Tumor Necrosis Factor-alpha (TNF-α), Interleukin-1 beta (IL-1β), and IL-6, while modulating apoptosis by downregulating BAX and upregulating Bcl-2, preventing caspase-3 activation.

Other therapeutics have also demonstrated nephroprotective effects when used concomitantly with cisplatin, including ammonium tetrathiomolybdate [49]; β-hydroxybutyrate [50]; diallyl trisulfide [51]; dihydroartemisinin [52]; omega-3-6-9 [53]; piracetam [54]; sodium thiosulfate [55]; and thrombomodulin [56].

Regarding ototoxicity, cisplatin induces hearing loss in 40%–60% of adults, including 18% who experience severe hearing loss, while in children, more than 70% are affected [57]. A recent study found that the cumulative incidence of cisplatin-induced hearing loss three years after treatment initiation was 75% in children under five years old, compared to 48% in adult patients [58].

Due to these severe side effects, other platinum drugs have been developed and approved for clinical use, including carboplatin and oxaliplatin (Table 1). Thousands of additional platinum-based molecules have been studied globally, some of which have been approved for clinical use in specific countries. These compounds generally act similarly to cisplatin by interacting with DNA [59].

Carboplatin, developed after cisplatin, is used to treat a range of cancers including ovarian, lung, certain head-and-neck cancers, and some metastatic breast cancer. It enters cells via transporters such as Ctr1, ATP7A, and ATP7B, as well as by simple diffusion. Once inside, carboplatin induces the expression of miR-145 and p53, and binds to DNA, causing deformation and initiating apoptosis [60].

A 2019 study comparing the genotoxic effects of cisplatin and carboplatin in cultured human lymphocytes found that both drugs significantly increased chromosomal aberrations, but cisplatin caused a higher frequency of sister-chromatid exchange, likely due to its higher reactivity and faster DNA binding kinetics compared to carboplatin [61]. However, a recent meta-analysis of 20 studies involving 4468 participants demonstrated that carboplatin is superior to cisplatin in treating early triple-negative breast cancer (TNBC), showing better disease-free survival (DFS), overall survival (OS), and pathological complete response (pCR), supporting the use of carboplatin for early TNBC [62]. Another meta-analysis, which included 11,049 patients, demonstrated that carboplatin, combined with dual HER2 blockade (pertuzumab + trastuzumab) plus docetaxel, was more effective than the same regimen without carboplatin in neoadjuvant treatment for HER2-positive breast cancer [63]. A third meta-analysis, conducted on 2111 patients with advanced or metastatic urothelial carcinoma (AMUC), demonstrated that cisplatin and carboplatin were similar to immune checkpoint inhibitor (ICI) monotherapy in terms of OS and pCR, but both platinum-based drugs had better objective response rates (ORR) than ICI therapy [64].

Nephrotoxicity observed with cisplatin occurs less frequently with carboplatin, with rates of 20%–30% for cisplatin *vs*. 10%–15% for carboplatin. This difference is likely due to structural changes in carboplatin, which result in less accumulation and thus reduced nephrotoxicity [65]. However, two recent studies evaluating a total of 362 patients found no significant difference in nephrotoxicity between cisplatin and carboplatin treatments [66,67]. Another study suggest that the risk of nephrotoxicity increased with age, particularly in patients treated with cisplatin compared to carboplatin, due to the natural decline in nephron number and size with age, which makes older individuals more susceptibility to nephrotoxicity [65].

Oxaliplatin was approved for clinical use by the FDA in 2002 and is indicated for cancers where resistance to cisplatin or carboplatin has developed. It is primarily used in the treatment of colorectal cancer, often in combination with other drugs such as 5-fluorouracil and leucovorin (FOLFOX regimen), FOLFOX plus irinotecan (FOLFOXIRI regimen); or oxaliplatin combined with capecitabine (CAPEOX regimen); among others [60].

The mechanism of oxaliplatin’s entry into the cell is similar to cisplatin, primarily through passive diffusion or via the CTR1 transporter [60]. Once inside, unlike cisplatin or carboplatin, oxaliplatin inhibits RNA Pol I, leading to rRNA silencing and nucleolar disruption, likely mediated by the DNA damage response kinases Ataxia Telangiectasia Mutated (ATM) and ATR serine/threonine kinase (ATR) [68].

Although oxaliplatin has fewer side effects than cisplatin, it can cause a neuropathy known as oxaliplatin-induced peripheral neurotoxicity (OIPN), which may lead to treatment interruption, though the condition can be reversible upon cessation of treatment. OIPN is thought to arise from DNA damage in sensory neurons, dysfunction of voltage-gated ion channels, increased pro-inflammatory response in certain neurons and peripheral nerves, oxidative stress from DNA adducts in neurons, and eventual neuronal apoptosis [69].

As noted, oxaliplatin is the treatment of election for colorectal cancer, often in combination with other drugs. A recent meta-analysis involving 795 patients demonstrated that the FOLFOX regimen was superior to fluorouracil plus leucovorin (IFL) or even oxaliplatin combined with irinotecan (IROX), in terms of median time to progression, response rate, and median survival time for the treatment of metastatic colorectal cancer [70]. Another meta-analysis, which included 4571 patients, compared regimens containing oxaliplatin and irinotecan for treating metastatic colorectal cancer, finding no statistically significant differences between the two treatment groups in terms of OS, progression-free survival (PFS), or ORR [71].

A recent study from the National Surgical Adjuvant Breast and Bowel Project (NSABP), known as the R-04 trial (NCT00058474), evaluated oxaliplatin’s toxicity in 1132 rectal cancer patients. These patients were randomized into four groups: 5-FU alone (n = 277), 5-FU + oxaliplatin (n = 286), capecitabine alone (n = 283), and capecitabine plus oxaliplatin (n = 286). The study found that oxaliplatin in combination with either 5-FU or capecitabine was less tolerable than either chemotherapy alone [72], suggesting that a careful balance between tumor control and side effects must be considered.

Next to the development of cisplatin (first generation), carboplatin, and oxaliplatin (second generation), a third generation of platinum-based drugs was developed. Among them, is nedaplatin (Table 1), which retains chemical similarities to cisplatin and carboplatin but offers improved anticancer and fewer side effects. Developed to enhance the water solubility of cisplatin and reduce its adverse effects while maintaining efficacy, nedaplatin is currently used clinically in Japan. It has demonstrated notable anticancer activity against cervical, small- and non-small cell lung, breast, ovarian, and testicular cancers, although it has not yet been widely accepted globally [73]. A study evaluating hypersensitivity to nedaplatin in 31 patients with carboplatin hypersensitivity showed a response rate of 71.4% in the nedaplatin-treated group, compared to 30.0% in the no-treated group. Only one patient in the nedaplatin group experienced a hypersensitivity reaction, demonstrating that nedaplatin is both safe and effective in this context [74].

Lobaplatin (Table 1) is another third-generation platinum-based drug, which appears to have lower nephro- and ototoxicity than its predecessors. Currently, it is only available in China. Lobaplatin was tested in bladder cancer cell lines T24 and 5637, showing IC_50_ values of 11.62 µg/mL and 9.61 µg/mL at 24 h, respectively. Flow cytometry revealed that lobaplatin induced apoptosis in 31.25% of T24 cells, compared to 6.25% in the control group, and 14.3% in 5637 cells, compared to 2.5% in the control. The study indicated that apoptosis was mediated by the regulation of Bcl-2 and BAX expression and inhibition of the PI3K/Akt signaling pathway [75].

Heptaplatin (Table 1), another third-generation platinum-based drug, was developed in South Korea and approved by the Korean FDA in 1999. Heptaplatin forms DNA adducts, altering transcription and replication processes, thereby inducing apoptosis [76]. A recent article explored supramolecular chemotherapy using heptaplatin and cucurbit-7uril (heptaplatin-CB7) to treat colorectal cancer cells. The results showed that heptaplatin-CB7 induced a notable percentage of early apoptosis in HCT116 and HT29 cells, and induced an inhibitory response in the G1 phase of the cell cycle in these cells [77].

The last third-generation platinum-based drug discussed here is miriplatin (Table 1), designed and synthesized in Japan for the treatment of unresectable hepatocellular carcinoma. Due to its poor solubility in water and organic solvents, miriplatin is unsuitable for intravenous administration [78]. In a recent study, miriplatin was encapsulated in a liposomal formulation called lipomiriplatin (LMPt) and tested in human pancreatic cancer cell lines (AsPC-1, BxPC-3, MIA-PaCa-2, PANC-1, and SU.86.86). The inhibitory effect of LMPt on these cancer cells was 2.70 to 16.74 times greater than that of miriplatin alone, with IC_50_ values ranging from 0.28 to 19.81 μmol/L. In comparison, oxaliplatin, used as a control, showed IC_50_ values greater than 75 μmol/L, indicating that LMPt is more effective than oxaliplatin. Additionally, EdU (a thymidine analog) was used to evaluate LMPt’s ability to inhibit DNA replication and cell proliferation, demonstrating superior activity compared to oxaliplatin in pancreatic cancer cells. *In vivo*, LMPt was tested in an AsPC-1 mouse xenograft model, where it exhibited a 31.36% inhibitory effect, approximately eight times greater than of miriplatin alone [78].

Despite their promising results, including chemical stability, low toxicity, and potent anticancer activity, even in cisplatin-resistant cancer cells, nedaplatin, lobaplatin, heptaplatin, and miriplatin have not been used or evaluated in Western countries. Economic factors may play a role in limiting their entry into Western healthcare markets, though the exact reasons remain unclear.

### Ruthenium compounds

Ruthenium-based molecules represent a large family of compounds with unique properties that make them promising candidates for cancer treatment. These properties include the ability to slowly exchange ligands with biologically important molecules like glutathione and certain proteins, and the capacity to exist in multiple oxidation states—Ru(II), Ru(III), and Ru(IV)—under physiological conditions. This allows some ruthenium complexes to be administered as a prodrug in the Ru(III) or Ru(IV) states and then reduced to the more reactive Ru(II) state in the tumor environment, thereby enhancing their cancer effectiveness [79,80]. Additionally, ruthenium can be transported throughout the body by plasma proteins such as albumin and transferrin, facilitating its distribution [81]. Once in circulation, ruthenium complexes can enter cells through transferrin receptors or death receptors, triggering apoptosis [82]. Some ruthenium complexes are internalized via endocytosis of transferrin receptors, while others may enter cells through passive diffusion [83].

Regarding the mechanism of action of ruthenium complexes, they can bind to DNA, disrupting DNA replication and RNA transcription, and can also stably bind to the G-quadruplex structure of telomeric DNA, interfering with telomerase activity. Additionally, ruthenium complexes can inhibit topoisomerases, which play key roles in DNA metabolism, leading to the initiation of apoptosis [80,84]. Recent studies have demonstrated that certain ruthenium complexes, such as [Ru(dip)_2_(PPβC)]PF_6_ (Table 2) and [Ru(phen)_2_(PPβC)]PF_6_, accumulate in mitochondria and inhibit mitochondrial DNA topoisomerase I, thereby inducing caspase-mediated apoptosis [85]. Another study highlighted a novel Ru(II) complex, [Ru(U)_2_(H_2_O)_2_]Cl_3_ (U = 5,6-Diamino-1,3-dimethylpyrimidine-2,4(1H,3H)-dione), which induces apoptosis by downregulating nuclear topoisomerase I expression [86]. Other studies have reinforced the observation that ruthenium complexes preferentially inhibit DNA topoisomerase I in cancer cells [87,88]. Similar results were shown with a thiomaltol-based [Ru(II)]PF_6_ complex, which has been shown to induce apoptosis in breast cancer cells by inhibiting DNA topoisomerase IIα, with a particular affinity for the DNA-binding pocket of the enzyme [89].

**Table 2:** The table represents some examples of the anti-cancer activity of ruthenium-based compounds

Structure	Ruthenium compounds	*In vitro* or *in vivo* activity	Mechanism of action	Reference
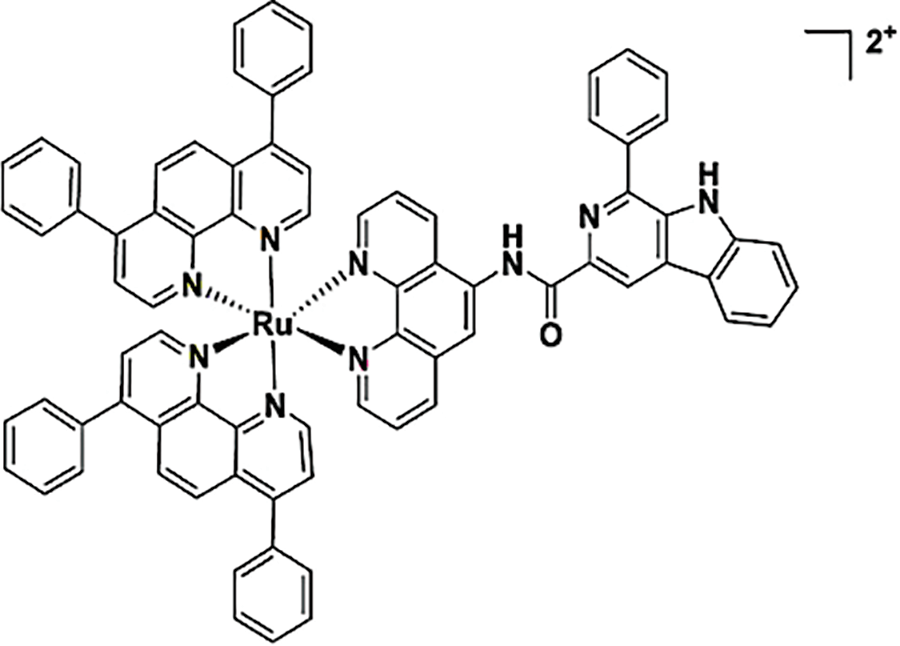	[Ru(dip)_2_(PPβC)]PF_6_	It exhibits cytotoxic activity against A549, HeLa, HepG2 and MCF-7 cancer cells	Inhibits mitochondrial DNA topoisomerase I, thereby inducing caspase-mediated apoptosis	[85]
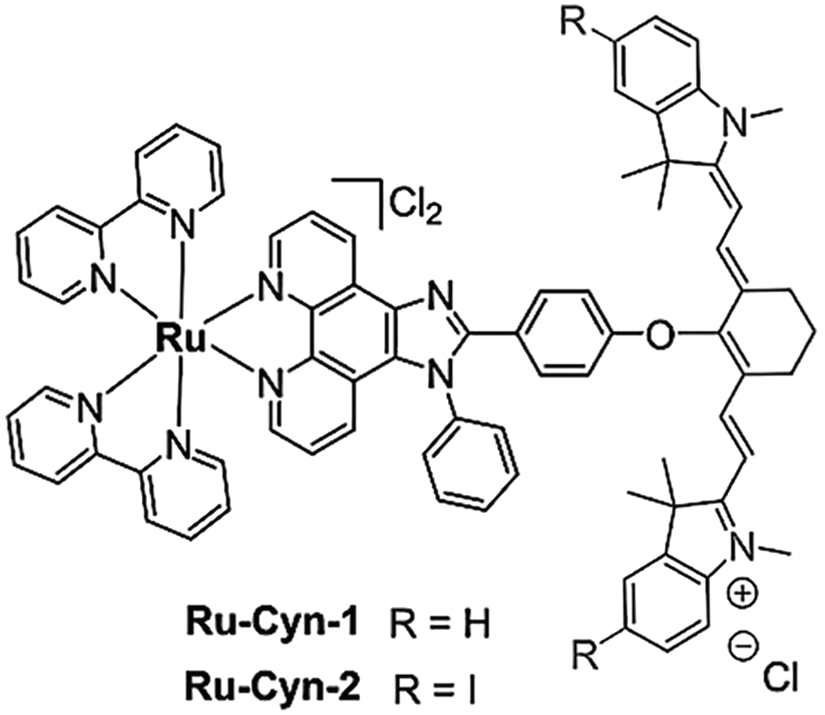	Ru-Cyn-1	Induces a 106-fold increase in cytotoxicity when photoactivated in colon cancer CT26 cells	It accumulates preferentially in the mitochondria and is able to promote the generation of both Type I and Type II ROS	[93]
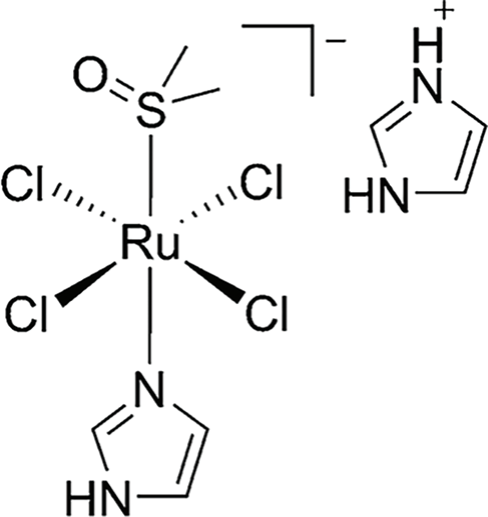	NAMI-A	Has been evaluated in patients with NSCLC	It can inhibit the formation of new blood vessels, inhibiting tumor metastasis. In addition, integrin α5β1 has also been proposed as a mechanism of action	[103]
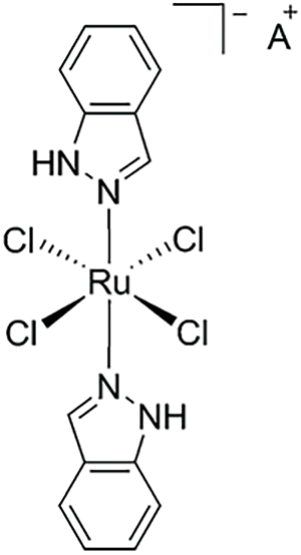	A^+^ = Na^+^ KP1339 A^+^ = IndH^+^ KP1019	It exhibits cytotoxicity in various types of tumor cells *in vitro*, including colorectal and ovarian cancer cells. Shows affinity for transport proteins such as transferrin, facilitating its cellular distribution.	It affects intracellular ROS levels, induces apoptosis through mitochondria or the MAPK/P38 pathway, and blocks the cell cycle in the G2/M phase.	[103]
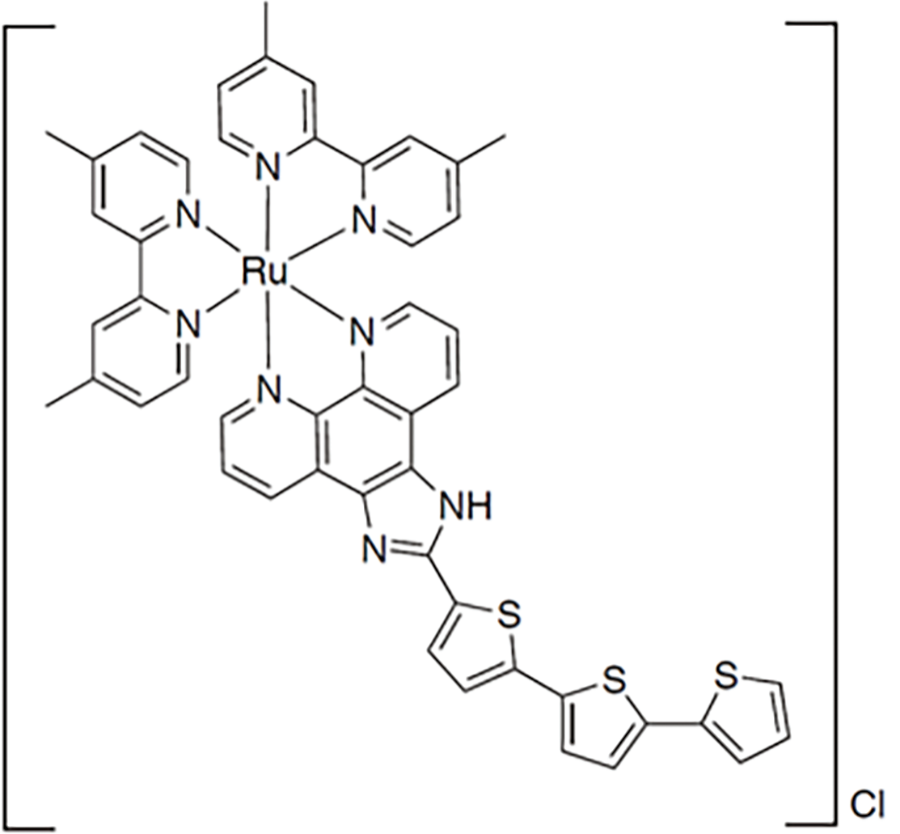	TLD-1433	Has been evaluated in patients with non-muscle-invasive bladder cancer (NMIBC)	Is activated with light activation, generating cytotoxic singlet oxygen and radical oxygen species, leading to cell death.	[103]

One of the most remarkable characteristics of ruthenium compounds is their ability to be photoactivated by light of specific wavelengths. This opens up possibilities for the development of ruthenium-based drugs for photodynamic therapy or photoactivated chemotherapy. In these therapies, the molecules can be selectively activated in the tumor area, thereby minimizing damage to healthy tissues. Over the past two years, several photoactivable ruthenium compounds have been developed for cancer treatment. For example, [Ru(phen-PPh_3_)_2_(1-Py-βC)](PF_6_)_4_ and [Ru(phen)_2_(1-Py-βC)](PF_6_)_2_, were shown to increase cytotoxicity by 50-100 times upon photoactivation in breast cancer MDA-MB-231 cells [90]. Other ruthenium complexes, such as cis-[Ru(dcbpyH)_2_(PTAH)_2_]Cl_2_, cis-[Ru(bpy)_2_(PTA)_2_]Cl_2_ and trans-[Ru(bpy)_2_(PTA)_2_](CF_3_SO_3_)_2_, exhibited 4- to 10-fold increase in cytotoxicity upon photoactivation in lung cancer A549 and prostate adenocarcinoma PC-3 cells [91]. Complexes Ru1−Ru5 of general formula [Ru(phen)_2_(N^∧^N′)]^2+^, being CH3 (Ru1), F (Ru2), CF_3_ (Ru3), NO_2_ (Ru4), and N(CH_3_)_2_ (Ru5) substituents in the phenyl ring, showed a 7- to 15-fold increase in cytotoxicity in cervical cancer HeLa cells and a 7- to 29-fold increase in cytotoxicity in A375 melanoma cells upon photoactivation [92]. Furthermore, the molecule Ru-Cyn-1 (Table 2) demonstrated a 106-fold increase in cytotoxicity when photoactivated in colon cancer CT26 cells [93].

At the molecular level, several genes are affected by ruthenium complexes. For example, the ruthenium complex RXC has been shown to downregulate the expression of genes encoding the chaperone Hsp90 in HCT116 colorectal cancer cells, along with downstream effectors of Hsp90, including Akt1, Akt (pS473), mTOR (pS2448), 4EBP1 (pT36/pT45), GSK-3β (pS9) and NF-κB p65 (pS529). This suggests that RXC induces cell death through inhibition of the AKT/mTOR pathway [94]. Similar results were observed with a ruthenium complex bound to 5-fluorouracil (Ru/5-FU), which inhibited the expression of Akt1 and Akt (pS473), mTOR (pS2448), S6 (pS235/pS236), 4EBP1 (pT36/pT45), GSK-3β (pS9) and NF-κB p65 (pS529) in HCT116 cells, further indicating inhibition of the Akt/mTOR pathway as a mechanism of action [95]. Another compound, RuZ2, demonstrated an IC_50_ of 4.05 μM in ovarian carcinoma SKO3CR cells. RuZ2 upregulated beclin-1, PINK1, Parkin, cleaved-caspase-3, caspase-9, and cytochrome c, while downregulating FNUDC1 and p62, suggesting a mechanism of cell death via mitophagy related-apoptosis [96]. Similarly, the ruthenium complex Ru-UCN1 was found to induce apoptosis in AGS cells by overexpressing p53, PUMA, and caspase-3 [97]. Another ruthenium compound, [Ru(η^6^-anethole)(en)X]PF_6_I, also induced significant overexpression of pro-apoptotic genes as caspase-3, PUMA, and DIABLO in AGS cells, supporting a mechanism of apoptosis similar to that seen with other ruthenium compounds [98]. Continuing this trend, another ruthenium compound, named complex-6, was shown to activate caspase-9 in dose- and time-dependent manner, along with up-regulation of BAX and cleaved-caspase-3, and downregulation of Bcl-2 in NCI-H460 lung cancer cells. This suggests that complex-6 induces caspase-mediated apoptosis through the intrinsic mitochondrial pathway in these cells [99]. A ruthenium biochanin-A compound has also been shown to induce apoptosis and cell cycle arrest in A549 lung cancer cells by activating caspase-3, while downregulating PI3K, TNF-α, TGF-β, and PPARγ in a dose-dependent manner [100]. Interestingly, this same compound was tested *in vivo* in Balb/c mice and was found to upregulate p53 and caspase-3 expression, while downregulating Bcl-2 in lung tissue [94]. Additionally, another ruthenium compound, a dendrimer named CRD13, was tested in Balb/c mice injected with 4T1 breast cancer cells. After 28 days of treatment with CRD13, tumor size decreased by nearly 50% compared to untreated control animals, and the ruthenium dendrimer did not affect the weight of mice [101].

An interesting ruthenium compound, NKP-1339, has undergone both preclinical and clinical testing. The biodistribution of this molecule was evaluated at different times in Balb/c mice bearing CT26 allograft (a murine colon cancer cell line). The study revealed that NKP-1339 initially accumulated in the serum within the first 4 h, after which its concentration gradually decreased. The kidneys and liver also accumulated the compound, likely due to their role in excretion and clearance. Only trace amounts of ruthenium were found in the brain, bones, and muscles up to 72 h post-administration. While ruthenium concentrations decreased in all organs over time, the concentration remained elevated in the kidneys. Notably, the tumor tissue exhibited higher ruthenium concentration at 24, 48, and 72 h compared to the initial 4 h, indicating preferential accumulation in the tumor over time [102].

To date, only a few ruthenium compounds have progressed to clinical trials as antitumor agents, including NAMI-A, KP1019, KP1339, and TLD1433 (all four in Table 2) [103]. These trials, which are primarily in Phases I or II, aim to determine maximal tolerable dose, dose-limiting toxicities, antitumor efficacy, as well as pharmacokinetics and pharmacodynamics of these ruthenium compounds. Details of these clinical studies will be covered in a separate section.

### Copper compounds

Copper is an essential metal involved in various redox processes and has emerged as a promising candidate for the development of new anticancer drugs. Copper can damage DNA, arrest the cell cycle, and induce apoptosis in tumor cells. Additionally, copper depletion has been shown to suppress angiogenesis, thereby inhibiting the neovascularization tumor of tumors [104,105].

Copper is primarily ingested in the form of Cu(II), which must be processed by intestinal cells for use by the body. These cells contain reductases on their surface, that reduce Cu(II) to Cu(I), facilitated by divalent metal transporters 1 (DMT1), while the copper transporter protein (CTR1) helps Cu(I) enter the cell. Once inside, it binds to the antioxidant chaperone protein 1 (ATOX1), which directs copper to the transporter proteins ATP7A and ATP7B. Copper is then bound to ceruloplasmin, which distributes it to various organs and tissues [106].

Intracellular copper concentrations must be tightly regulated, as elevated levels can lead to cytotoxicity and apoptosis. The FDX1 protein plays a crucial role in copper-induced cell death by acting as a reducing factor that converts Cu(II) to the more toxic Cu(I) form, increasing intracellular copper concentrations. This leads to the rapid generation of reactive oxygen species (ROS), which causes oxidative stress and ultimately triggers apoptosis [107,108].

Elesclomol (Table 3), a copper ionophere, has demonstrated high efficiency in inhibiting colorectal cancer cells (SW480) by inducing copper overload. This molecule degrades ATP7A, preventing copper from being transported and eliminated from the cells. The resulting accumulation of ROS diminishes the transporter responsible for GSH production, ultimately leading to ferroptosis in the cells [109]. Ghasemi et al. [110] studied the effect of copper nanoparticles (CuNPs) on the same SW480 colon cancer cells, showing that CuNPs induce cytotoxicity and ROS-mediated apoptosis, this was accompanied by increased expression of proapoptotic proteins BAX and P53 and decreased expression of Bcl-2. Similarly, in the breast cancer cell line (MCF-7), CuNPs were found to increase the expression of P53 and BAX, and the activation of caspase-8 and -9, suggesting that CuNPs trigger apoptosis through both intrinsic and extrinsic pathways [111].

**Table 3:** The table represents some examples of the anti-cancer activity of copper-based compounds

Structure	Cooper compound	*In vitro* and *in vivo* activity	Mechanism of action	Reference
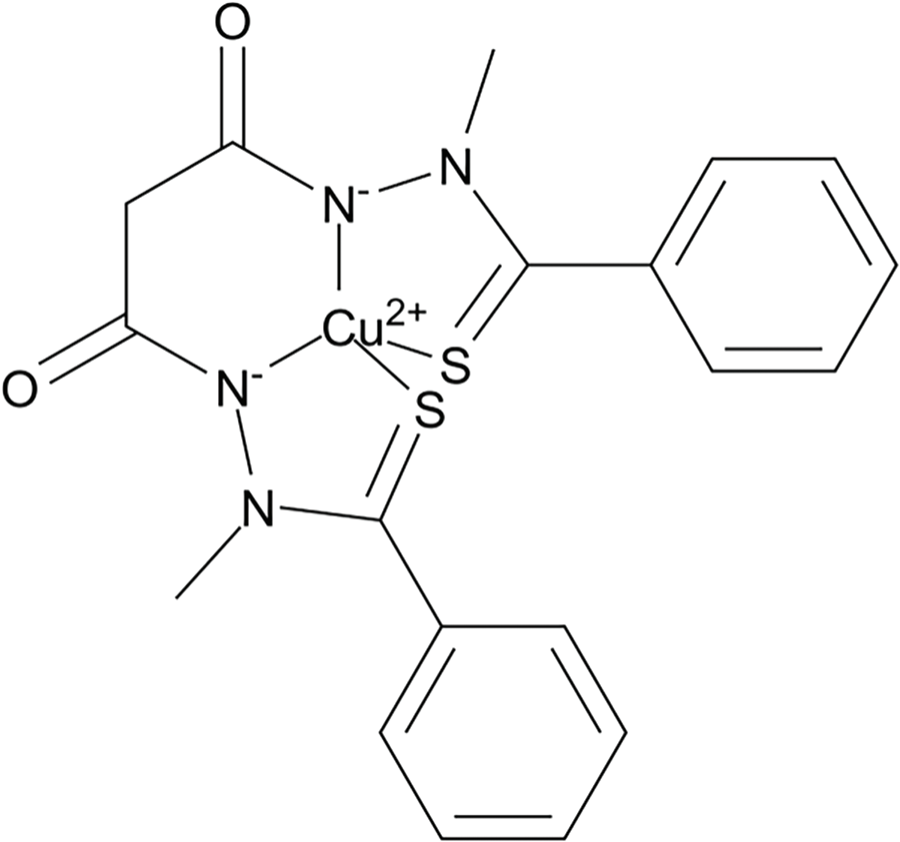	Elesclomol-Cu^2+^ complex	Shows high efficiency in inhibiting colorectal cancer cells (SW480)	It induces degradation of ATP7A, preventing copper elimination. Increases ROS and decreases GSH, leading to ferroptosis.	[109,110]
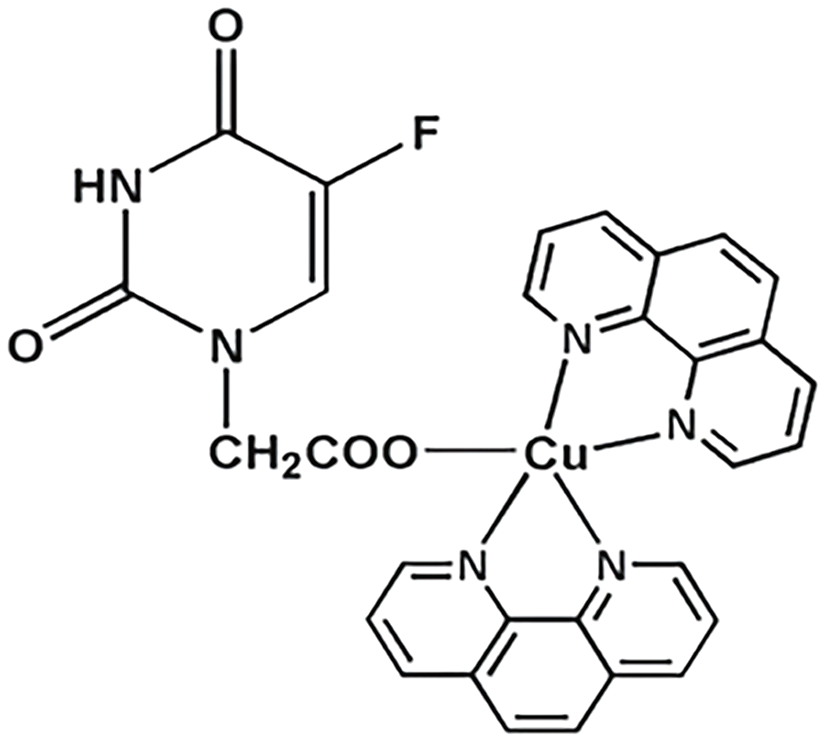	[Cu(phen)_2_L1] BF_4_H_2_O(Z9)	It induces cytotoxic effects on colon cancer cells (HCT116) and triple-negative breast cancer cells (TNBC)	Induction of apoptosis in cancer cells through interaction with DNA and oxidative stress	[113]
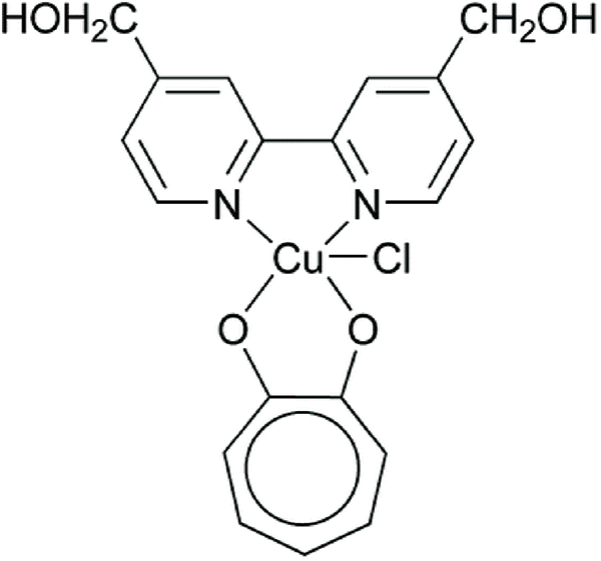	Cu(Trop)Cl	Inhibition of cell proliferation in colon cancer cells. Reduces cell migration	Interaction with DNA, causing genetic damage. Generation of oxidative stress due to its redox-active nature	[116]

In addition to inducing oxidative stress, copper, like other metals, can interact with DNA and cause genetic damage due to its reactive and redox-active nature, which allows it to bond with various functional groups. Researchers have explored these characteristics by binding copper with ligands such as 5-fluoracyl and phenanthrolines to enhance their anticancer properties [112,113]. For example, two Cu(II) complexes [Cu (bpy)_2_L1] BF_4_·CH_3_OH and [Cu(phen)_2_L1] BF_4_·H_2_O, with L1 = 5-fluorouracil-1-yl acetic acid (Table 3), were studied as anticancer agents against colon cancer cells (HCT116) and triple-negative breast cancer cells (TNBC) (MDA-MB-231), demonstrating cytotoxic effects on both cell lines. This study also showed that DNA binding by Cu(II) is enhanced when conjugated to 5-fluorouracil [113]. In an effort to reduce the side effects of copper-based molecules, researchers have formulated ligands that are selective for tumor cells. One example is the development of curcumin-derived ligands, as curcumin itself has demonstrated anticancer properties. Curcumin-copper complexes have shown cytotoxic effects in breast cancer cells, with IC_50_ ranging between 2.3–7.1 μM, significantly lower than carboplatin (IC_50_ = 359.3 μM). Moreover, these compounds were tested in human umbilical vein endothelial cells (HUVECs), showing a higher IC_50_, which indicates lower cytotoxicity compared to carboplatin, making them promising alternatives with selective toxicity for cancer cells [114].

Recent research has explored the effects of copper compounds on other types of cancer cells. Machado et al. [115] tested the cytotoxic activity of copper in OVCAR3 ovarian and PC3 prostate cancer cells, finding high anticancer activity in both cell lines. In PC3 cells, these copper compounds were 7 to 22 times more active than cisplatin. When investigating the mechanism of cell death in OVCAR3 cells, the induction of apoptosis was indicated as the main mechanism, ruling out oxidative stress as a contributing factor.

Additionally, two copper complexes, [Cu(Trop)Sac] and [Cu(Trop)Cl] (Table 3), which contain a tropolone structure along with saccharin or chlorine, have been studied for their anticancer effects. These compounds were tested in colon cancer cells, where the chlorine-containing molecule [Cu(Trop)Cl] exhibited antiproliferative effects, a property not observed with the saccharin-containing molecule [Cu(Trop)Sac]. However, in migration assays, both molecules inhibited cancer cell movement, with [Cu(Trop)Sac] demonstrating a stronger effect. This suggests that both [Cu(Trop)Sac] and [Cu(Trop)Cl], hold potential as therapeutic agents for reducing invasiveness and metastasis in colon cancer [116].

### Gold compounds

Since the FDA approved the gold compound auranofin (Table 4) for the treatment of rheumatoid arthritis in the 1980s, interest in gold compounds for therapeutic applications has significantly increased [117]. Gold(I) and gold(III) ions can be conjugated to various atoms such as nitrogen, phosphorus, selenium, carbon, and others, resulting in complexes with diverse geometric configurations. The multiple oxidation states, high electronegativity, and electron affinity of gold make its complexes promising candidates for cancer therapy. These complexes can modulate immune responses, inhibit key enzymes involved in cell proliferation, and induce apoptosis [118–120].

**Table 4:** The table represents some examples of the anti-cancer activity of gold-based compounds

Structure	Gold compound	*In vitro* activity	Mechanism of action	Reference
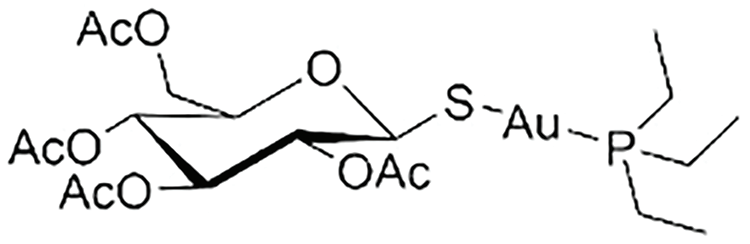	Auranofin	Inhibits the proliferation of TNBC cell line MDA-MB-231	Inhibits TrxR, leading to increased intracellular ROS levels, causing oxidative stress and apoptosis. Affects DNA polymerases and induces apoptosis	[117]
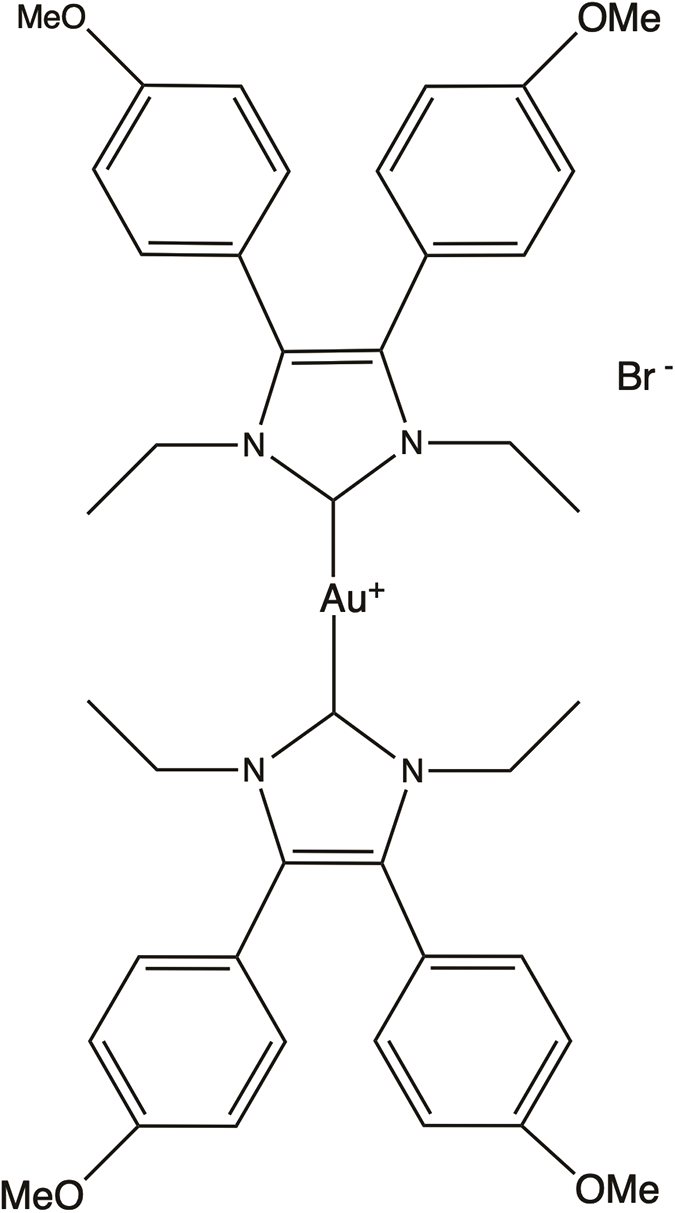	Au4BC	Dose-dependent reduction in cell viability of HCC (Huh7) and TNBC (MDA-MB-231) cell lines	Induction of cell death by modulating intracellular ROS levels. Increases γ-H2AX levels, suggesting DNA damage and activation of repair mechanisms	[127]
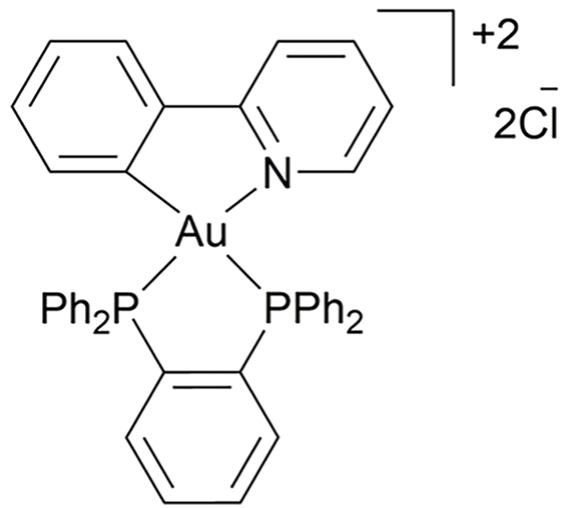	AuPhos-89	Disruption of pathways related to inflammation and mitochondrial function in TNBC cell line MDA-MB-231	Induction of apoptosis by modulating oxidative phosphorylation and redox pathways, impacting mitochondrial metabolism	[128]
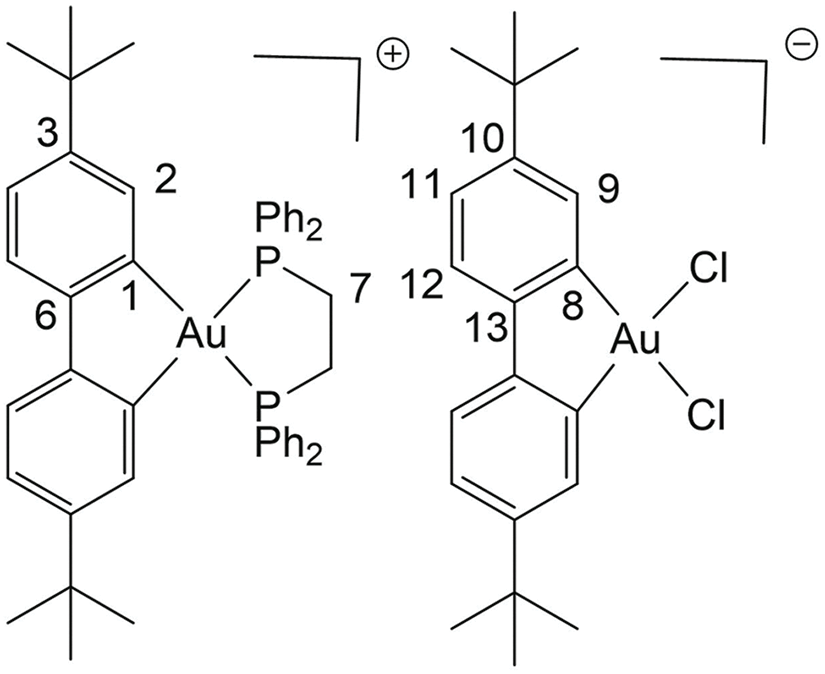	[(C^C)Au(Cl)(L^L)(Cl)Au(C^C)]	It exhibits strong antiproliferative activity against TNBC cell line MDA-MB-231 and ovary A2780 cells	Not clear	[130]

Gold(I) compounds have a lower affinity for DNA but show a strong preference for sulfhydryl, thiol, and selenocysteine groups in important protein targets [121,122]. For instance, gold(I) interacts with the thiol and selenol groups of thioredoxin reductases (TrxRs), inhibiting their normal function. Since TrxRs are critical for maintaining cellular redox homeostasis, their inhibition leads to elevated intracellular ROS levels, oxidative stress, and ultimately, apoptosis [123]. Gold(I) compounds also bind to the sulfhydryl groups of DNA polymerases, impeding their action and reducing the acuracy of DNA replication, triggering programmed cell death. While gold(III) compounds also induce apoptosis, they do so through different mechanisms. In physiological conditions, ligands such as diphosphines, terpyridines, dithiolates, and diamines stabilize gold(III). Notably, terpyridine-based gold(III) complexes form strong, irreversible bonds with DNA, a key mechanism underlying their potent antiproliferative effects in cancer cells [124].

Studies have shown that [C^N]Au(III) cyclometalated gold compounds with a norbornane scaffold and secondary diamines exhibit antiproliferative effects on ovarian cancer cells. These compounds demonstrate high toxicity toward glycolysis-dependent cells compared to cells reliant on oxidative phosphorylation, suggesting that, unlike other metal-based drugs, they do not cause damage to mitochondria [125]. Other research has explored gold compounds as a means of overcoming cisplatin resistance in ovarian cancer cells. In a recent study, the cytotoxicity of four molecules [Au(mnt)_2_] ^−^, [Au(i-mnt)_2_] ^−^, [Au(cdc)_2_] ^−^ and [Au(qdt)_2_] ^−^ was evaluated in ovarian cancer cell lines A2780 and A2780cisR (cisplatin resistant). After 48 h of exposure, the IC_50_ values ranged from 0.9 to 5.5 μM in both cancer cell lines, demonstrating strong cytotoxic effects [126].

Another study conducted on MDA-MB-231 cells, a model of TNBC, revealed that the gold compound Au4BC (Table 4) exhibited cytotoxic effects, causing a dose-dependent reduction in cell viability. This was accompanied by a significant increase in γ-H2AX levels, a marker of DNA damage that is recruited to double-strand break sites to facilitate DNA repair, as well as an increase in ROS levels. These findings suggest that Au4BC may induce cell death by modulating ROS levels within the cells [127]. Similarly, AuPhos-89 (Table 4), a gold(III)-bisphosphine complex, demonstrated alterations in biological pathways related to inflammation and mitochondrial function in MDA-MB-231 cells. AuPhos-89 appears to induce apoptosis by modulating oxidative phosphorylation and redox pathways, impacting mitochondrial metabolism. *In vivo* studies using a murine model of TNBC showed that AuPhos-89 significantly inhibits tumor growth, positioning it as a promissory compound for cancer treatment [128]. Other molecules, such as alkylgold(III) compounds, have also shown cytotoxic effects in MDA-MB-231 and MCF-7 breast cancer cell lines, particularly when irradiated with UV light. The cytotoxicity of these photoreactive alkylgold(III) compounds is significantly enhanced in the presence of light, suggesting a light-dependent activation mechanism. This ability to selectively activate compounds using UV light offers greater precision in drug delivery, potentially reducing systemic toxicity and improving therapeutic efficacy [129].

Giuso et al. [130] evaluated the anticancer potential of four binuclear biphenyl organogold(III) complexes, of general formula [(C^C)Au(Cl)(L^L)(Cl)Au(C^C)] (Table 4), across various cellular models. Notably, one of these complexes demonstrated potent antiproliferative effects in A549 lung cancer, MDA-MB-231 breast cancer, and A2780 ovarian cancer cells, with IC_50_ values of 0.13, 0.20 and 0.07 μM, respectively. The most surprising finding was that these compounds significantly lower cytotoxicity against non-cancer cells compared to cisplatin, which is a highly desirable feature in anticancer compounds.

Despite the growing body of research on gold complexes, further studies are needed, particularly in preclinical models, followed by clinical trials to fully assess their therapeutic potential.

Some examples of the anti-cancer activity of gold-based compounds can be seen in Table 4.

### Palladium compounds

Palladium(II) and platinum(II) species show certain structural and thermodynamic similarities, making palladium(II) complexes an interesting alternative to platinum-based drugs. Recent studies have shown that several palladium(II) complexes exhibit greater bioactivity compared to platinum(II)-based drugs. Moreover, these palladium(II) complexes have demonstrated low toxicity and high specificity [131]. Palladium complexes could bind strongly with DNA via the minor groove and show good binding affinity for bovine serum albumin (BSA), which may facilitate their targeted delivery to tumors [132–134].

Palladium-based nanomaterials also exhibit strong absorption in the near-infrared (NIR) region, with high photothermal conversion efficiency, photothermal stability, and biocompatibility. These properties make palladium-based compounds plausible candidates for photothermal therapy, where the drugs are activated by light to selectively target cancer cells [135].

Palladium(II) complexes with thioamide ligands have shown potential as anticancer agents. The presence of aromatic N-donor motifs enhances their activity, while sulfur offers diverse coordination possibilities for metal centers, contributing to their effectiveness [136,137]. Palladium complexes are thought to be less toxic than cisplatin. For example, the median lethal dose (LD50) of cisplatin administered orally to rats is approximately 270 mg/kg, whereas the LD50 for palladium is over 2700 mg/kg, indicating tenfold lower toxicity [136].

Palladium is capable of inducing mitochondrial dysfunction through the increase of mitochondrial ROS, alteration of membrane potential, and release of cytochrome c. This mitochondrial disturbance can alter oxidative phosphorylation in cells treated with palladium complexes, thereby affecting cell viability and increasing the likelihood of apoptosis [138].

One study evaluated the cytotoxicity of various palladium(II) triphenylphosphine complexes of thioamides against the human prostate cancer cell line, PC3. In particular, the complexes [Pd(Tu)_2_(PPh_3_)_2_]Cl_2_, [Pd(Dmtu)_2_(PPh_3_)_2_]Cl_2_, and [Pd(Mpm)_2_(PPh_3_)_2_]Cl_2_ exhibited notable cytotoxic activity, with IC_50_ values of 18.30, 5.80, and 8.17 µM, respectively, which are desirable for antitumor agents [139].

Another study on breast cancer cell lines, MCF7, MC4L2, and 4T1, demonstrated that the palladium compound L^2^PdCl, containing thioamide ligands, was capable of inhibiting cell proliferation with an IC_50_ value of 20 µM across all cell lines after 48 h of treatment. Two other palladium compounds were also evaluated, but their IC_50_ values ranged from 40 to 100 µM [136].

A recent article evaluated a photoactivable palladium compound, PdL (Table 5), in tumor xenografts of human skin melanoma (A375) in nude mice. PdL showed minimal inhibition of tumor growth in the dark; however, when the animals were irradiated with green light 12 h after drug injection, tumor growth was strongly inhibited. Additionally, PdL exhibited low cytotoxicity in healthy organs, indicating its potential for selective cancer treatment [140].

**Table 5:** The table represents some examples of the anti-cancer activity of other metal-based compounds

Structure	Name	*In vitro* and *in vivo* activity	Mechanism of action	Reference
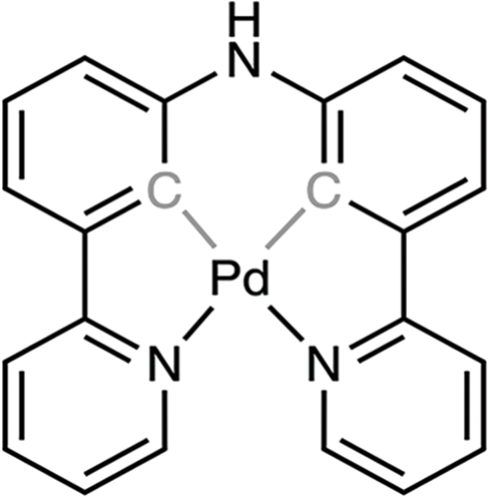	PdL	Inhibits tumor growth in melanoma xenografts when irradiated with light. Low cytotoxicity in healthy organs	Photoactivable molecule, induces the production of ROS in cancer cells after ligth irradiation.	[140]
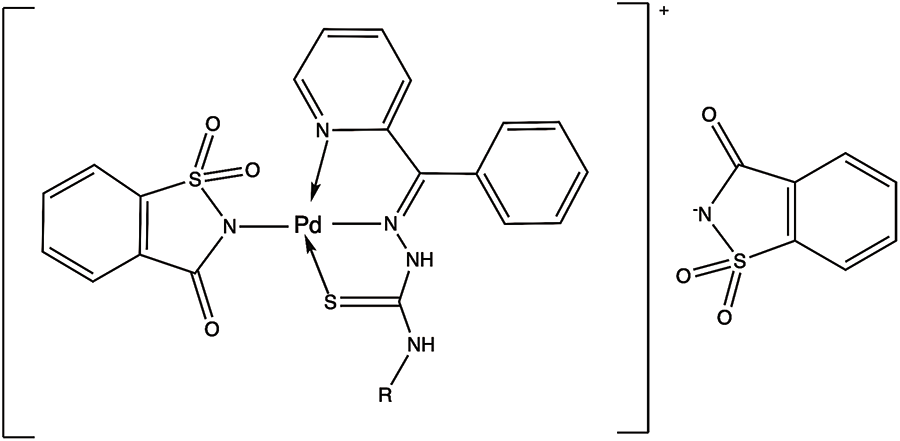	9a	It exhibits antiproliferative activity in A549 and Spc-A1 lung cancer cell lines	It induces apoptosis by activate caspase-3 and -9, and downregulate Bcl-2 expression.	[141]
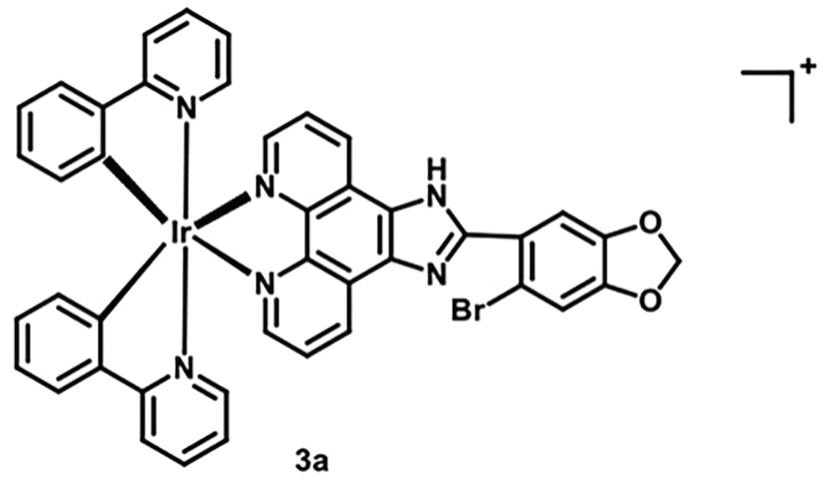	[Ir(ppy)_2_(BDIP)](PF_6_)	It exhibits antiproliferative activity in A549 lung cancer cells	Photoactivable molecule, affects mitochondrial membrane potential by releasing cytochrome c, and activating caspase-3, ultimately resulting in apoptosis.	[154]
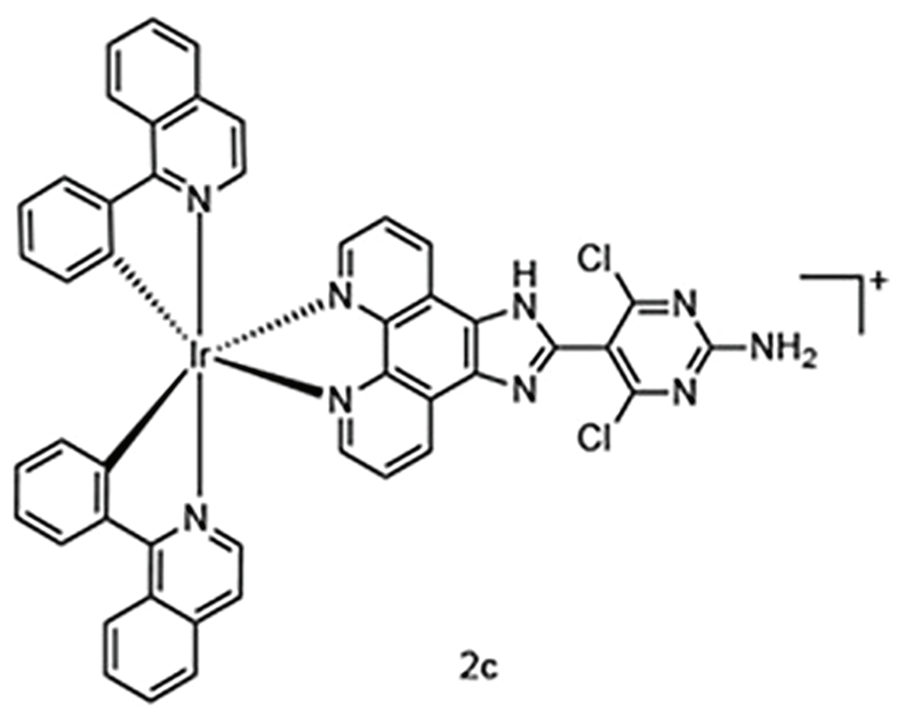	[Ir(piq)_2_Cl]_2_	It exhibits antiproliferative activity in A549 lung cell cancer, B16 melanoma, and HCT116 colorectal cancer cells	It affects mitochondrial function, elevating ROS and inducing the release of cytochrome c, which causes ferroptosis and pyroptosis.	[157]
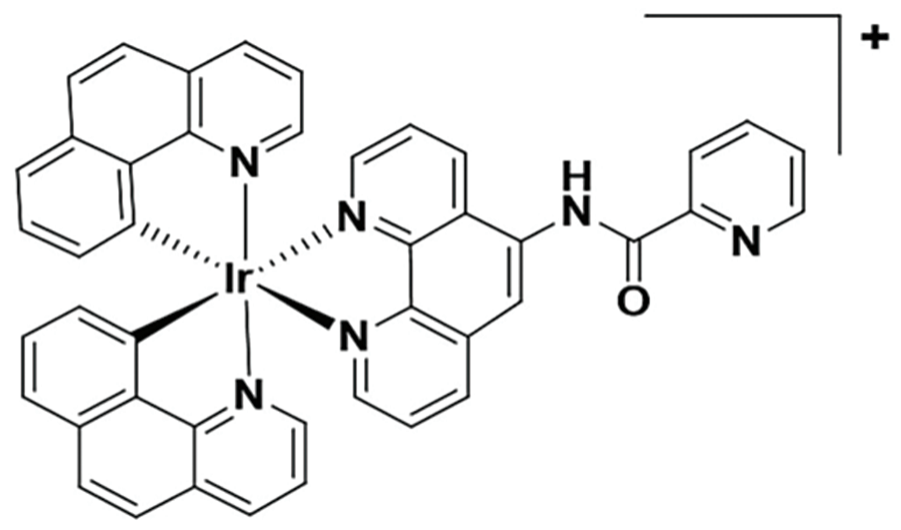	[Ir(bzq)_2_PPA]PF_6_	It exhibits antiproliferative activity in A549 lung cell cancer	It affects mitochondrial function, elevating ROS and inducing the release of cytochrome c. Additionally, it induces cell cycle arrest in the S phase and apoptosis through the AKT-mediated signaling pathway.	[158]

Another recent study evaluated the anticancer potential of several palladium-based drugs *in vitro* and *in vivo* in lung adenocarcinoma models. Palladium compounds were tested in A549 and Spc-A1 lung cancer cell lines, with IC_50_ values ranging from 2 to 26 µM, while in the normal cell line 293T, the IC_50_ values ranged from 119 to 172 µM. Notably, a compound called 9a (Table 5) showed IC_50_ values of 2.4 and 7.28 µM in A549 and Spc-A1 cell lines, respectively, compared to 130.56 µM in 293T cells, demonstrating selective toxicity towards cancer cells. Flow cytometry analysis confirmed that compound 9a induced apoptosis in lung cancer cells. Furthermore, 9a activated caspase-3 and -9, and downregulated the antiapoptotic gene Bcl-2. In *in vivo* studies, animals treated with compound 9a showed a six-fold decrease in tumor weight, highlighting its potential as a promising anticancer agent [141].

A coumarin-palladium(II) complex, designated as C1, was evaluated against the pancreatic carcinoma cell line PANC-1. The IC_50_ value for C1 in these cells was 3.39 µM. Flow cytometry showed that treatment with 5 μM of the C1 complex induces 10% necrosis and 20% late apoptosis in the cells. In comparison, the control drug doxorubicin caused four times the level of cell death due to necrosis. Additionally, C1 significantly upregulated BAK and downregulated Bcl-2 in PANC-1 cells, markers of apoptosis. Finally, in an *in vivo* zebrafish-PANC-1 xenograft model, treatment with 0.5 μM of C1 resulted in a tumor mass reduction of approximately 60%, demonstrating the compound’s potent anticancer activity [142].

Some examples of the anti-cancer activity of palladium-based compounds can be seen in Table 5.

### Zinc compounds

Zinc plays an essential role in nutrition and in human health, with a recommended daily intake of 6.7 to 15 mg per day [143]. The FDA has approved zinc oxide (ZnO) as a safe metal oxide, which is less toxic than many other metals and exhibits low reactivity with active pharmaceutical ingredients. The pharmacokinetic properties and biodistribution of ZnO nanoparticles *in vivo* can be enhanced using nanotechnology; studies have shown that orally administered ZnO tends to accumulate in the liver, kidneys, and lungs. The primary route of ZnO excretion is through feces, while smaller particles are excreted via urine [144].

In cancer patients, serum zinc levels often decrease significantly, disrupting zinc metabolism. This reduction has been observed in a variety of cancers, including breast, ovarian, gastrointestinal, lung, thyroid, and esophageal cancers [145].

ZnO nanoparticles have been associated with anticancer activity, likely through the generation of ROS, which activates the release of apoptotic factors from mitochondria, ultimately inducing cell death. Additionally, ZnO nanoparticles can interact with the negatively charged environment of tumor cells, a characteristic of the Warburg effect. ZnO nanoparticles release Zn(II) ions into the cell nucleus, where they can interact with DNA and RNA, disrupting processes such as replication and transcription [146].

The first evidence of the antiproliferative effects of zinc in cancer was reported in the late 1990s when Liang and colleagues found that physiological levels of zinc inhibited cell growth in dose-dependent prostate cancer cell lines LNCaP and PC-3. Their study demonstrated that zinc induces G2/M phase cell cycle arrest through the overexpression of p21 [147].

Since then, several *in vivo* and *in vitro* studies have confirmed the effectiveness of zinc compounds as anticancer agents and tumor suppressors, primarily through the modulation of growth-related genes and signaling pathways [148].

Previous studies using MTT assays, cell clone formation, Hoechst staining, and flow cytometry have demonstrated that exposure of PC-3 prostate cancer cells to zinc can enhance the antitumor activity of paclitaxel (an anti-microtubule taxane) by nearly 50%. This effect is believed to be mediated through the mitochondria-dependent apoptosis pathway. Additionally, an increase in the activation of caspase-3 and caspase-9 was observed, along with a reduction in the Bcl-2/BAX expression ratio, all hallmark characteristics of apoptosis [149]. Subsequent studies further confirmed that the combination of paclitaxel and zinc affects cell proliferation in a dose- and time-dependent manner in PC3 and DU145 prostate cancer cells. This drug combination has also been shown to inhibit prostate cancer cell invasion and migration by downregulating the expression of TWIST1, a transcription factor involved in embryonic development and cancer progression [150].

In more recent research, ZnO nanoparticles were synthesized in combination with *Rubus fairholmianus* root extract (named RFZnO) to investigate their synergistic cytotoxic effects on breast cancer MCF-7 cells. Rubus species have been used in traditional medicine due to their diverse pharmacological properties, and previous studies have reported the *in vitro* cytotoxic effects of *R. fairholmianus* on colorectal, breast, and lung cancers. The results demonstrated that RFZnO nanoparticles significantly increased cytotoxicity in MCF-7 cells (5.22-fold) compared to untreated controls. Additionally, there was a marked increase (4.57-fold) in cytochrome c release in the treated cells. RFZnO nanoparticles also induced the overexpression of BAX and p53, while downregulating Bcl-2 at both the mRNA and protein levels. These findings suggest that RFZnO nanoparticles induce apoptosis in breast cancer cells through a mitochondria-mediated, caspase-dependent apoptotic pathway [151].

As mentioned earlier, various natural extracts can reduce the adverse effects of metal-based drugs while also enhancing their anticancer activities. In one study, the cytotoxic and antitumor effects of zinc oxide nanoparticles (ZnO NPs), prepared with pure curcumin (Green-ZnO-NPs), were evaluated on the breast cancer cell line MCF-7. The results showed a significant reduction in MCF-7 cell viability, with IC_50_ values of 23.54 and 20.53 μg/mL at 24 and 48 h, respectively, demonstrating an increase in activity over time. Curcumin itself did not show anticancer activity before 24 h of treatment, indicating that it enhanced the anticancer activity of the zinc nanoparticles.

A recent study also assessed the effect of ZnO, among other compounds, on the efficacy of cisplatin in MCF-7 cells. The IC_50_ value for cisplatin alone was 9.1 μg/mL, whereas the IC_50_ for the ZnO/cisplatin complex was 2.12 μg/mL at 24 h, more than 4 times lower than cisplatin alone. Furthermore, flow cytometry analysis showed that the ZnO/cisplatin complex induced higher early apoptosis (67.02%) compared to the cisplatin control (53.59%). Additionally, a chitosan-ZnO/cisplatin complex exhibited a higher level of late apoptosis (57.75%) compared to the cisplatin control (35.96%), demonstrating that ZnO can significantly enhance the anticancer activity of cisplatin [152].

Although the precise mechanism of ZnO nanoparticle cytotoxicity remains unclear, the most widely accepted theory involves the intracellular release of Zn(II) ions, along with generation of ROS [153]. This mechanism, together with changes in the expression of pro- and anti-apoptotic genes, likely contributes to the observed cytotoxic effects.

### Iridium compounds

Recently, iridium(III) compounds have shown a great potential as anticancer drugs, since they can interact with DNA and induce alteration of mitochondrial function, in addition to enhancing the generation of reactive ROS, inducing endoplasmic reticulum stress and apoptosis [154,155]. Another interesting feature of iridium(III) is its ability to absorb and emit light, making it a good candidate for targeted anticancer photodynamic therapy [156]. In this regard, a recent article evaluated the photoactivity of two iridium(III) compounds, [Ir(ppy)_2_(BDIP)](PF_6_) (Table 5) and [Ir(ppy)_2_(MDIP)](PF_6_), which were tested on A549 lung cancer cells. When cells were treated for 48 h, the IC_50_ values were 4 and 26.8 μM, respectively. However, when cells were treated with these iridium compounds and exposed to white light for 48 h, the IC_50_ values were 0.7 and 1.8 μM, respectively, which represent a considerable improvement in the activity of these compounds. The IC_50_ of cisplatin in these cells was 6.6 μM, indicating that these photoactivable iridium compounds could be excellent drugs for targeted therapy [154].

Another recent study performed in A549 lung cell cancer, B16 melanoma, and HCT116 colorectal cancer cells, showed that [Ir(piq)_2_Cl]_2_ (Table 5) has potent antiproliferative activity with IC_50_ values of 2.2, 2.5, and 2.5 μM, respectively, while the IC_50_ for cisplatin in these cells was 6.1, 28.8, and 15.3 μM, respectively. Again, these iridium compounds show significantly greater antitumor activity compared to cisplatin in the treated cells. Furthermore, it was determined that this iridium compound is capable of affecting mitochondrial function opening the mitochondrial permeability transition pore, elevating ROS, and inducing a release of cytochrome c, which causes ferroptosis and pyroptosis. Finally, this iridium compound was tested *in vivo* in Balb/c nude mice xenotransplanted with A549 cells, demonstrating that it was able to induce a tumor inhibition rate of 34.04% with the treatment of 3.2 mg/kg and of 58.58% with 5.0 mg/kg of drug [157].

In other studies, the A549 cells were treated with the iridium compound [Ir(bzq)_2_PPA]PF_6_ (Table 5) showing an IC_50_ of 1.6 μM, while the IC_50_ values for cisplatin and oxaliplatin were 18.6 and 13.5 μM, respectively. This indicates that the iridium compound increases cytotoxicity in A549 cells more than 10-fold compared to traditional platinum-based chemotherapy [158]. This increased activity opens up an interesting opportunity to develop more drugs of this type that allow the use of low doses with greater potential.

Some examples of the anti-cancer activity of iridium-based compounds can be seen in Table 5.

### Clinical assays

#### Clinical trials with platinum drugs

Despite the increasing exploration of alternative therapies, hundreds of studies published last year alone highlight ongoing clinical trials using platinum-based drugs, typically in combination with other treatments, for cancer therapy.

In particular, cisplatin is widely used as a chemotherapy drug due to its good results and low cost, however, many cancer patients show partial or no response when treated with cisplatin for a long time and develop resistance. This cisplatin resistance is partly due to the hypoxic tumor microenvironment (TME), which results in elevated expression of HIF-1α, which is involved in the induction of angiogenesis, changes in the glucose metabolism, cell proliferation, inhibition of apoptosis, invasion and metastasis, finally leading to an adaptation to the hypoxic TME [159].

Platinum drugs combined with etoposide and anti–PD-L1 therapies currently serve as the standard first-line treatment for extensive-stage small cell lung cancer (ES-SCLC). In a study involving 457 patients, 227 received tislelizumab (a human anti–PD–1 antibody) alongside chemotherapy (180 patients with etoposide plus carboplatin and 47 with etoposide plus cisplatin), while 230 patients received placebo plus chemotherapy (181 with etoposide plus carboplatin and 49 with etoposide plus cisplatin). The results showed that tislelizumab, when combined with platinum-etoposide chemotherapy, significantly reduced the risk of death and improved PFS, ORR, and duration of response (DoR). These findings suggest that tislelizumab plus chemotherapy could become the new first-line treatment for ES-SCLC patients [160].

In another recent study involving 1214 patients with non-small cell lung cancer (NSCLC), the efficacy of avelumab (a human anti-PD-L1 IgG1 antibody) was evaluated in 688 patients, compared with 526 patients receiving platinum-based doublet chemotherapy. The differences in OS and PFS between the two groups were not statistically significant, indicating that platinum-based therapy remains the best option for patients with high-expression PD-L1+ NSCLC [161].

Additionally, for patients with EGFR-mutated metastatic NSCLC, the combination of nivolumab (another human anti-PD-1 antibody) with platinum-based doublet chemotherapy (cisplatin or carboplatin) was evaluated in 141 patients, compared with platinum chemotherapy alone in 143 patients. No significant differences were found in PFS, ORR, or DoR between the groups, further confirming that platinum-based chemotherapy is a viable and effective option for treating metastatic NSCLC [162].

In an interesting study, selenium yeast, a feed additive, was evaluated for its potential to prevent adverse effects related to platinum-based combination therapy in patients with malignant tumors. Selenium yeast, a selenium supplement, is primarily used to promote the well-being of patients with tumors, and cardiovascular, or cerebrovascular diseases. In this study involving 86 patients, 43 received platinum treatment (cisplatin or carboplatin) combined with selenium yeast at a dose of 200 μg daily, while the other 43 received platinum treatment without selenium yeast. The results indicated that patients supplemented with selenium yeast experienced a significant reduction in adverse reactions associated with platinum therapy. Benefits included improved appetite, prevention of weight loss, and significant pain relief, making selenium yeast a promising adjunct for managing the side effects of platinum-based chemotherapy without compromising its efficacy [163].

In Phases I/II study, the efficacy of dendritic cell (DC) vaccination combined with carboplatin/paclitaxel was evaluated in 5 patients with metastatic endometrial cancer (mEC). The results demonstrated that DC vaccination can be safely combined with carboplatin/paclitaxel chemotherapy in these patients, suggesting a potential new synergistic treatment option with promising outcomes [164].

In a retrospective study involving 198 patients with ovarian cancer, different chemotherapy regimens were evaluated. Of these, 92 patients (46.5%) received gemcitabine plus carboplatin, 76 patients (38.4%) received paclitaxel plus carboplatin, and 30 patients (15.2%) received gemcitabine plus cisplatin combined with bevacizumab. The group that received gemcitabine, cisplatin, and bevacizumab had the highest overall survival (OS) compared to the other groups, suggesting a potential benefit of this combination therapy [165].

#### Clinical trials with ruthenium drugs

In a Phase I study involving 31 patients with NSCLC, NAMI-A combined with gemcitabine was evaluated. Many patients experienced common adverse effects, including fatigue, nausea, vomiting, and diarrhea. Out of 15 evaluable patients, only 1 experienced partial remission, suggesting that NAMI-A’s efficacy may not surpass that of gemcitabine alone [166]. In 2009, the ruthenium-based drug KP1019 was administered to 7 patients with various solid tumors to assess its pharmacokinetics. No dose-limiting toxicity was observed, likely due to the low solubility of the drug. KP1019 showed low clearance and renal excretion was responsible for eliminating only a small fraction of ruthenium. Biliary excretion was suggested as the primary route of elimination for KP1019. However, the study did not evaluate the drug’s effect on the tumors [167]. A more recent study evaluated the safety and tolerability of the photoactivable ruthenium compound TLD-1433 in 6 patients with non–muscle-invasive bladder cancer (NMIBC). The study showed that TLD-1433 was well tolerated, with no serious adverse events. Three patients were treated with a dose of 0.70 mg/cm^2^ of TLD-1433. Drug concentrations in the urine were measured at 60.1 ng/mL and 0.6 ng/mL at 24 and 72 h, respectively, and the drug was undetectable after 72 h. Of these three patients, two achieved complete responses at 180 days, although one patient developed metastatic disease at 138 days. Overall, TLD-1433 demonstrated good tolerability and safety, with potential efficacy [168].

Over the past ten years, no clinical trials have been conducted with KP1019, KP1339, or other non-platinum metal-based drugs in cancer patients.

## Conclusions

Given the increase in cancer worldwide, it has become necessary to find new drugs capable of inhibiting tumor growth and inducing apoptosis in cancer cells specifically, without affecting normal cells, thereby avoiding undesirable effects on patients. Metal-based drugs therefore appear to have considerable potential as novel therapeutic agents against cancer because they display wide chemical diversity and versatility, exist in different oxidation states, coordinate diverse types of ligands, exhibit redox activity, and react with important biomolecules in cells, such as proteins or DNA. Metallodrugs based on platinum, ruthenium, copper, gold, palladium, zinc, and iridium exhibit different mechanisms of action, target different components, and show varying toxicities, making them a promising avenue for overcoming drug resistance in cancer treatment.

Platinum-based drugs were the first metal-based molecules whose antiproliferative activity was described in the mid-1960s, particularly cisplatin, which was approved by the FDA in the late 1970s. Since then, the development of new drugs has progressed rapidly, with drugs such as carboplatin, oxaliplatin, nedaplatin, lobaplatin, heptaplatin, and miriplatin, now in clinical use in various countries. Unfortunately, other metal-based drugs have not been as successful, with only a few ruthenium compounds—such as NAMI-A, KP1019, KP1339, and TLD1433—reaching clinical studies, but only in Phases I or II. No recent clinical trials have been conducted with other metal-based drugs.

Various copper-based compounds have been tested on colon, breast, prostate, and ovarian cancer cells, demonstrating their ability to induce the activity of pro-apoptotic molecules such as p53, BAX, caspase-8 and -9, while inhibiting the expression of Bcl-2. Moreover, copper compounds have been shown to be more effective than platinum-based molecules in the studies reviewed.

Gold compounds appear to induce apoptosis through different mechanisms, either by binding to proteins involved in cell cycle control or directly interacting with DNA. Interestingly, some gold compounds, unlike other metal-based compounds, do not cause mitochondrial damage, while others are photoreactive, suggesting a light-dependent activation mechanism. These compounds can be activated by UV light, allowing for greater precision in drug efficacy.

This photoactivatable activity has also been observed in palladium and iridium compounds, which were tested on cancer cells, showing greater anticancer activity when irradiated with light. Similar results were observed in palladium compounds, which were tested on tumor xenografts in nude mice, showing higher inhibition of tumor growth when the animals were irradiated with light. Other palladium and iridium compounds have also been evaluated *in vivo*, showing a significant decrease in tumor weight in treated animals.

Zinc-based drugs have been combined with other drugs, such as paclitaxel and cisplatin, showing significantly higher activity compared to the drugs used alone.

Additionally, interesting studies combining natural compounds with metal-based drugs were reviewed, indicating that these not only improve antitumor activity but also reduce cytotoxicity in normal cells, which could potentially reduce side effects for patients in future applications of metal-based compounds.

The limitation of this review is the lack of clinical studies, so we only analyzed results on some platinum compounds. However, we did not find clinical studies with other metal-based compounds, which restricted the discussion on the topic. We hope that more metal-based complexes, beyond the well-known platinum-based drugs, can advance to clinical trials. The *in vitro* and *in vivo* results for these compounds are very promising, but there is a notable lack of pharmacokinetic and pharmacodynamic studies for many of these metallodrugs, which are highlighted in this review.

Advancements in these types of studies could provide new opportunities for millions of cancer patients worldwide.

## Data Availability

Not applicable.

## References

[1] Bray F, Laversanne M, Sung H, Ferlay J, Siegel RL, Soerjomataram I, et al. Global cancer statistics 2022: GLOBOCAN estimates of incidence and mortality worldwide for 36 cancers in 185 countries. CA Cancer J Clin. 2024;74(3):229–63. doi:10.3322/caac.v74.3. 38572751

[2] Ghosh S. Cisplatin: the first metal based anticancer drug. Bioorg Chem. 2019;88:102925. doi:10.1016/j.bioorg.2019.102925; 31003078

[3] Riccardi C, Piccolo M. Metal-based complexes in cancer. Int J Mol Sci. 2023;24(8):7289. doi:10.3390/ijms24087289; 37108457 PMC10138440

[4] Romani AMP. Cisplatin in cancer treatment. Biochem Pharmacol. 2022;206:115323. doi:10.1016/j.bcp.2022.115323; 36368406

[5] Beirne DF, Farkaš B, Donati C, Gandin V, Rozas I, Velasco-Torrijos T, et al. Novel design of dual-action Pt (IV) anticancer pro-drugs based on cisplatin and derivatives of the tyrosine kinase inhibitors imatinib and nilotinib. Dalton Trans. 2023;52(39):14110–22. doi:10.1039/D3DT02030D; 37747105

[6] Pernar Kovač M, Tadić V, Kralj J, Duran GE, Stefanelli A, Stupin Polančec D, et al. Carboplatin-induced upregulation of pan β-tubulin and class III β-tubulin is implicated in acquired resistance and cross-resistance of ovarian cancer. Cell Mol Life Sci. 2023;80(10):294. doi:10.1007/s00018-023-04943-0; 37718345 PMC11071939

[7] Sanchon-Sanchez P, Briz O, Macias RIR, Abad M, Sanchez-Martin A, Marin JJG, et al. Evaluation of potential targets to enhance the sensitivity of cholangiocarcinoma cells to anticancer drugs. Biomed Pharmacother. 2023;168:115658. doi:10.1016/j.biopha.2023.115658; 37832404

[8] Tsang ES, Aggarwal RR, Dhawan MS, Bergsland EK, Alvarez EA, Calabrese S, et al. A phase IB trial of the PI3K inhibitor Alpelisib and weekly cisplatin in patients with solid tumor malignancies. Cancer Res Commun. 2022;2(7):570–6. doi:10.1158/2767-9764.CRC-22-0028; 36923283 PMC10010328

[9] Thaklaewphan P, Wikan N, Potikanond S, Nimlamool W. Oxyresveratrol enhances the anti-cancer effect of cisplatin against epithelial ovarian cancer cells through suppressing the activation of protein Kinase B (AKT). Biomolecules. 2024;14(9):1140. doi:10.3390/biom14091140; 39334906 PMC11430010

[10] Chen RL, Wang Z, Huang P, Sun CH, Yu WY, Zhang HH, et al. Isovitexin potentiated the antitumor activity of cisplatin by inhibiting the glucose metabolism of lung cancer cells and reduced cisplatin-induced immunotoxicity in mice. Int Immunopharmacol. 2021;94:107357. doi:10.1016/j.intimp.2020.107357; 33715980

[11] Motie FM, Soltani Howyzeh M, Ghanbariasad A. Synergic effects of DL-limonene, R-limonene, and cisplatin on AKT, PI3K, and mTOR gene expression in MDA-MB-231 and 5637 cell lines. Int J Biol Macromol. 2024;280:136216. doi:10.1016/j.ijbiomac.2024.136216; 39362430

[12] Lee KC, Wu KL, Chang SF, Chang HI, Chen CN, Chen YY. Fermented ginger extract in natural deep eutectic solvent enhances cytotoxicity by inhibiting NF-κB mediated CXC chemokine receptor 4 expression in oxaliplatin-resistant human colorectal cancer cells. Antioxid. 2022;11(10):2057. doi:10.3390/antiox11102057; 36290780 PMC9598626

[13] Mbemi AT, Sims JN, Yedjou CG, Noubissi FK, Gomez CR, Tchounwou PB. Vernonia calvoana shows promise towards the treatment of ovarian cancer. Int J Mol Sci. 2020;21(12):4429. doi:10.3390/ijms21124429; 32580345 PMC7352360

[14] Li Y, Wu Y, Xia Q, Zhao Y, Zhao R, Deng S. Platycodon grandiflorus enhances the effect of DDP against lung cancer by down regulating PI3K/Akt signaling pathway. Biomed Pharmacother. 2019;120:109496. doi:10.1016/j.biopha.2019.109496; 31610427

[15] Wen L, Hu J, Zhang J, Yang J. Phenylethanol glycosides from Herba Cistanche improve the hypoxic tumor microenvironment and enhance the effects of oxaliplatin via the HIF-1α signaling pathway. Mol Med Rep. 2021;24(1):517. doi:10.3892/mmr.2021.12156; 34013363 PMC8160477

[16] Sedky NK, Abdel-Kader NM, Issa MY, Abdelhady MMM, Shamma SN, Bakowsky U, et al. Co-delivery of Ylang Ylang oil of cananga odorata and oxaliplatin using intelligent PH-sensitive lipid-based nanovesicles for the effective treatment of triple-negative breast cancer. Int J Mol Sci. 2023;24(9):8392. doi:10.3390/ijms24098392; 37176099 PMC10179110

[17] Wang C, Chen C, Chen X, Luo J, Su Y, Liu X, et al. Identification of genes predicting chemoresistance and short survival in ovarian cancer. Transl Cancer Res. 2024;13(8):4354–71. doi:10.21037/tcr. 39262489 PMC11385244

[18] Lucaciu RL, Hangan AC, Sevastre B, Oprean LS. Metallo-drugs in cancer therapy: past, present and future. Molecules. 2022;27(19):6485. doi:10.3390/molecules27196485; 36235023 PMC9572156

[19] Czarnomysy R, Radomska D, Szewczyk OK, Roszczenko P, Bielawski K. Platinum and palladium complexes as promising sources for antitumor treatments. Int J Mol Sci. 2021;22(15):8271. doi:10.3390/ijms22158271; 34361037 PMC8347039

[20] Kacsir I, Sipos A, Bényei A, Janka E, Buglyó P, Somsák L, et al. Reactive oxygen species production is responsible for antineoplastic activity of osmium, ruthenium, iridium and rhodium half-sandwich type complexes with bidentate glycosyl heterocyclic ligands in various cancer cell models. Int J Mol Sci. 2022;23(2):813. doi:10.3390/ijms23020813; 35054999 PMC8776094

[21] Geisler H, Harringer S, Wenisch D, Urban R, Jakupec MA, Kandioller W, et al. Systematic study on the cytotoxic potency of commonly used dimeric metal precursors in human cancer cell lines. ChemistryOpen. 2022;11(7):e202200019. doi:10.1002/open.202200019; 35212190 PMC9278098

[22] Łomzik M, Błauż A, Tchoń D, Makal A, Rychlik B, Plażuk D. Development of half-sandwich Ru, Os, Rh, and Ir complexes bearing the Pyridine-2-ylmethanimine bidentate ligand derived from 7-Chloroquinazolin-4(3H)-one with enhanced antiproliferative activity. ACS Omega. 2024;9(16):18224–37. doi:10.1021/acsomega.3c10482; 38680348 PMC11044151

[23] Selim MS, Kassem AB, El-Bassiouny NA, Salahuddin A, Abu El-Ela RY, Hamza MS. Polymorphic renal transporters and cisplatin’s toxicity in urinary bladder cancer patients: current perspectives and future directions. Med Oncol. 2023;40(2):80. doi:10.1007/s12032-022-01928-0; 36650399 PMC9845168

[24] Ciarimboli G. Membrane transporters as mediators of Cisplatin and side effects. Anticancer Res. 2014;34(1):547–50; 24403515

[25] Tan WJT, Vlajkovic SM. Molecular characteristics of cisplatin-induced ototoxicity and therapeutic interventions. Int J Mol Sci. 2023;24(22):16545. doi:10.3390/ijms242216545; 38003734 PMC10671929

[26] Lukanović D, Herzog M, Kobal B, Černe K. The contribution of copper efflux transporters ATP7A and ATP7B to chemoresistance and personalized medicine in ovarian cancer. Biomed Pharmacother. 2020;129:110401. doi:10.1016/j.biopha.2020.110401; 32570116

[27] Arnesano F, Natile G. Interference between copper transport systems and platinum drugs. Semin Cancer Biol. 2021;76:173–88. doi:10.1016/j.semcancer.2021.05.023; 34058339

[28] Jeon J, Lee S, Kim H, Kang H, Youn H, Jo S, et al. Revisiting platinum-based anticancer drugs to overcome gliomas. Int J Mol Sci. 2021;22(10):5111. doi:10.3390/ijms22105111; 34065991 PMC8151298

[29] Zhang C, Xu C, Gao X, Yao Q. Platinum-based drugs for cancer therapy and anti-tumor strategies. Theranostics. 2022;12(5):2115–32. doi:10.7150/thno.69424; 35265202 PMC8899578

[30] Dasari S, Njiki S, Mbemi A, Yedjou CG, Tchounwou PB. Pharmacological effects of cisplatin combination with natural products in cancer chemotherapy. Int J Mol Sci. 2022;23(3):1532. doi:10.3390/ijms23031532; 35163459 PMC8835907

[31] Minerva, Bhat A, Verma S, Chander G, Jamwal RS, Sharma B, et al. Cisplatin-based combination therapy for cancer. J Cancer Res Ther. 2023;19(3):530–6. doi:10.4103/jcrt.jcrt_792_22; 37470570

[32] Fang CY, Lou DY, Zhou LQ, Wang JC, Yang B, He QJ, et al. Natural products: potential treatments for cisplatin-induced nephrotoxicity. Acta Pharmacol Sin. 2021;42(12):1951–69. doi:10.1038/s41401-021-00620-9; 33750909 PMC8633358

[33] Bork T, Hernando-Erhard C, Liang W, Tian Z, Yamahara K, Huber TB. Cisplatin nephrotoxicity is critically mediated by the availability of BECLIN1. Int J Mol Sci. 2024;25(5):2560. doi:10.3390/ijms25052560; 38473806 PMC10931997

[34] Schofield J, Harcus M, Pizer B, Jorgensen A, McWilliam S. Long-term cisplatin nephrotoxicity after childhood cancer: a systematic review and meta-analysis. Pediatr Nephrol. 2024;39(3):699–710. doi:10.1007/s00467-023-06149-9; 37726572 PMC10817831

[35] McMahon KR, Lebel A, Rassekh SR, Schultz KR, Blydt-Hansen TD, Cuvelier GDE, et al. Acute kidney injury during cisplatin therapy and associations with kidney outcomes 2 to 6 months post-cisplatin in children: a multi-centre, prospective observational study. Pediatr Nephrol. 2023;38(5):1667–85. doi:10.1007/s00467-022-05745-5; 36260162

[36] El-Gohary RM, Ghalwash AA, Awad MM, El-Shaer RAA, Ibrahim S, Eltantawy AF, et al. Novel insights into the augmented effect of curcumin and liraglutide in ameliorating cisplatin-induced nephrotoxicity in rats: effects on oxidative stress, inflammation, apoptosis and pyroptosis via GSK-3β. Arch Biochem Biophys. 2023;749:109801. doi:10.1016/j.abb.2023.109801; 37884117

[37] Qiu CW, Chen B, Zhu HF, Liang YL, Mao LS. Gastrodin alleviates cisplatin nephrotoxicity by inhibiting ferroptosis via the SIRT1/FOXO3A/GPX4 signaling pathway. J Ethnopharmacol. 2024;319:117282. doi:10.1016/j.jep.2023.117282; 37802374

[38] Luo X, Xie D, Chen Z, Ji Q. Protective effects of ginsenosides in cisplatin-induced kidney injury: a systematic review, meta-analysis. Indian J Pharmacol. 2023;55(4):243–50. doi:10.4103/ijp.ijp_251_23; 37737077 PMC10657623

[39] Chen XC, Huang LF, Tang JX, Wu D, An N, Ye ZN, et al. Asiatic acid alleviates cisplatin-induced renal fibrosis in tumor-bearing mice by improving the TFEB-mediated autophagy-lysosome pathway. Biomed Pharmacother. 2023;165:115122. doi:10.1016/j.biopha.2023.115122; 37413899

[40] Alherz FA, Elekhnawy E, Selim HM, El-Masry TA, El-Kadem AH, Hussein IA, et al. Protective role of betulinic acid against cisplatin-induced nephrotoxicity and its antibacterial potential toward uropathogenic bacteria. Pharmaceuticals. 2023;16(8):1180. doi:10.3390/ph16081180; 37631096 PMC10458273

[41] Lee B, Kim YY, Jeong S, Lee SW, Lee SJ, Rho MC, et al. Oleanolic acid acetate alleviates cisplatin-induced nephrotoxicity via inhibition of apoptosis and necroptosis *in vitro* and *in vivo*. Toxics. 2024;12(4):301. doi:10.3390/toxics12040301; 38668524 PMC11054587

[42] Wang L, Xie Y, Xiao B, He X, Ying G, Zha H, et al. Isorhamnetin alleviates cisplatin-induced acute kidney injury via enhancing fatty acid oxidation. Free Radic Biol Med. 2024;212:22–33. doi:10.1016/j.freeradbiomed.2023.12.010; 38101584

[43] Shao YF, Tang BB, Ding YH, Fang CY, Hong L, Shao CX, et al. Kaempferide ameliorates cisplatin-induced nephrotoxicity via inhibiting oxidative stress and inducing autophagy. Acta Pharmacol Sin. 2023;44(7):1442–54. doi:10.1038/s41401-023-01051-4; 36658427 PMC10310756

[44] Gómez-Sierra T, Ortega-Lozano AJ, Rojas-Morales P, Medina-Reyes EI, Barrera-Oviedo D, Pedraza-Chaverri J. Isoliquiritigenin pretreatment regulates ER stress and attenuates cisplatin-induced nephrotoxicity in male Wistar rats. J Biochem Mol Toxicol. 2023;37(12):e23492. doi:10.1002/jbt.v37.12. 37561086 PMC13547121

[45] Lin SY, Chang CL, Liou KT, Kao YK, Wang YH, Chang CC, et al. The protective role of *Achyranthes aspera* extract against cisplatin-induced nephrotoxicity by alleviating oxidative stress, inflammation, and PANoptosis. J Ethnopharmacol. 2024;319:117097. doi:10.1016/j.jep.2023.117097; 37648176

[46] Ibrahim D, Halboup A, Al Ashwal M, Shamsher A. Ameliorative effect of olea europaea leaf extract on cisplatin-induced nephrotoxicity in the rat model. Int J Nephrol. 2023;2023:2074498. doi:10.1155/2023/2074498; 37497380 PMC10368505

[47] Mahmod II, Ismail IS, Normi YM, Chong SG. Protective effect of Clinacanthus nutans in cisplatin-induced nephrotoxicity on human kidney cell (PCS-400-010) elucidated by an LCMS-based metabolomics approach. Biomed Chromatogr. 2023;37(12):e5750. doi:10.1002/bmc.5750; 37778127

[48] Turan I, Canbolat D, Demir S, Kerimoglu G, Colak F, Turkmen Alemdar N, et al. The ameliorative effect of *Primula vulgaris* on cisplatin-induced nephrotoxicity in rats and quantification of its phenolic components using LC-ESI-MS/MS. Saudi Pharm J. 2023;31(9):101730. doi:10.1016/j.jsps.2023.101730; 37583754 PMC10424254

[49] Qi H, Shi H, Yan M, Zhao L, Yin Y, Tan X, et al. Ammonium tetrathiomolybdate relieves oxidative stress in cisplatin-induced acute kidney injury via NRF2 signaling pathway. Cell Death Discov. 2023;9(1):259. doi:10.1038/s41420-023-01564-1; 37491360 PMC10368633

[50] Tian R, Tang S, Zhao J, Hao Y, Zhao L, Han X, et al. β-hydroxybutyrate protects against cisplatin-induced renal damage via regulating ferroptosis. Ren Fail. 2024;46(1):2354918. doi:10.1080/0886022X.2024.2354918; 38757723 PMC11104694

[51] Jiang X, Zhu X, Liu Y, Zhou N, Zhao Z, Lv H. Diallyl trisulfide and its active metabolite allyl methyl sulfone attenuate cisplatin-induced nephrotoxicity by inhibiting the ROS/MAPK/NF-κB pathway. Int Immunopharmacol. 2024;127:111373. doi:10.1016/j.intimp.2023.111373; 38128310

[52] Luo Y, Zhang J, Jiao Y, Huang H, Ming L, Song Y, et al. Dihydroartemisinin abolishes cisplatin-induced nephrotoxicity *in vivo*. J Nat Med. 2024;78(2):439–54. doi:10.1007/s11418-024-01783-5; 38351420

[53] Abd Alhusen SK, Hasan AF. Evaluating the renoprotective effects of omega-3-6-9 against cisplatin-induced nephrotoxicity in mice. J Med Life. 2023;16(12):1756–9. doi:10.25122/jml-2023-0078; 38585532 PMC10994620

[54] El-Dessouki AM, Alzokaky AA, Raslan NA, Ibrahim S, Salama LA, Yousef EH. Piracetam mitigates nephrotoxicity induced by cisplatin via the AMPK-mediated PI3K/Akt and MAPK/JNK/ERK signaling pathways. Int Immunopharmacol. 2024;137:112511. doi:10.1016/j.intimp.2024.112511; 38909496

[55] Senguttuvan RN, Santiago NL, Han ES, Lee B, Lee S, Lin WC, et al. Impact of sodium thiosulfate on prevention of nephrotoxicities in HIPEC: an ancillary evaluation of cisplatin-induced toxicities in ovarian cancer. Ann Surg Oncol. 2023;30(13):8144–55. doi:10.1245/s10434-023-14216-6; 37710139 PMC10625947

[56] Yamamoto H, Ishida Y, Zhang S, Osako M, Nosaka M, Kuninaka Y, et al. Protective roles of thrombomodulin in cisplatin-induced nephrotoxicity through the inhibition of oxidative and endoplasmic reticulum stress. Sci Rep. 2024;14(1):14004. doi:10.1038/s41598-024-64619-y; 38890434 PMC11189513

[57] Wang X, Zhou Y, Wang D, Wang Y, Zhou Z, Ma X, et al. Cisplatin-induced ototoxicity: from signaling network to therapeutic targets. Biomed Pharmacother. 2023;157:114045. doi:10.1016/j.biopha.2022.114045; 36455457

[58] Meijer AJM, Li KH, Brooks B, Clemens E, Ross CJ, Rassekh SR, et al. The cumulative incidence of cisplatin-induced hearing loss in young children is higher and develops at an early stage during therapy compared with older children based on 2052 audiological assessments. Cancer. 2022;128(1):169–79. doi:10.1002/cncr.v128.1. 34490624

[59] McAdam AD, Batchelor LK, Romano-deGea J, Vasilyev D, Dyson PJ. Thermoresponsive carboplatin-releasing prodrugs. J Inorg Biochem. 2024;254:112505. doi:10.1016/j.jinorgbio.2024.112505; 38377623

[60] Forgie BN, Prakash R, Telleria CM. Revisiting the anti-cancer toxicity of clinically approved platinating derivatives. Int J Mol Sci. 2022;23(23):15410. doi:10.3390/ijms232315410; 36499737 PMC9793759

[61] Azab B, Alassaf A, Abu-Humdan A, Dardas Z, Almousa H, Alsalem M, et al. Genotoxicity of cisplatin and carboplatin in cultured human lymphocytes: a comparative study. Interdiscip Toxicol. 2019;12(2):93–7. doi:10.2478/intox-2019-0011; 32206030 PMC7071837

[62] Mason SR, Willson ML, Egger SJ, Beith J, Dear RF, Goodwin A. Platinum chemotherapy for early triple-negative breast cancer. Breast. 2024;75:103712. doi:10.1016/j.breast.2024.103712; 38492276 PMC10959715

[63] Villacampa G, Matikas A, Oliveira M, Prat A, Pascual T, Papakonstantinou A. Landscape of neoadjuvant therapy in HER2-positive breast cancer: a systematic review and network meta-analysis. Eur J Cancer. 2023;190:112885. doi:10.1016/j.ejca.2023.03.042; 37142539

[64] Mori K, Schuettfort VM, Yanagisawa T, Katayama S, Pradere B, Laukhtina E, et al. Reassessment of the efficacy of carboplatin for metastatic urothelial carcinoma in the era of immunotherapy: a systematic review and meta-analysis. Eur Urol Focus. 2022;8(6):1687–95. doi:10.1016/j.euf.2022.02.007; 35279408

[65] Van der Gaag S, Labots M, Swart EL, Crul M. Reducing renal function assessment prior to platinum-based chemotherapy: a real-world evaluation. Acta Oncol. 2024;63:169–74. doi:10.2340/1651-226X.2024.23960; 38597664 PMC11332549

[66] Fernandes ARDS, de Brito GA, Baptista AL, Andrade LAS, Imanishe MH, Pereira BJ. The influence of acute kidney disease on the clinical outcomes of patients who received cisplatin, carboplatin, and oxaliplatin. Health Sci Rep. 2022;5(1):e479. doi:10.1002/hsr2.479; 35036578 PMC8753493

[67] Choudhary R, Bundela MK, Bharang K. Comparison of renal functions evaluated by measured glomerular filtration rate in patients treated with cisplatin, carboplatin, and oxaliplatin. Cureus. 2023;15(3):e36549. doi:10.7759/cureus.36549; 37095803 PMC10121481

[68] Nechay M, Wang D, Kleiner RE. Inhibition of nucleolar transcription by oxaliplatin involves ATM/ATR kinase signaling. Cell Chem Biol. 2023;30(8):906–919.e4. doi:10.1016/j.chembiol.2023.06.010; 37433295 PMC10529435

[69] Cheng F, Zhang R, Sun C, Ran Q, Zhang C, Shen C, et al. Oxaliplatin-induced peripheral neurotoxicity in colorectal cancer patients: mechanisms, pharmacokinetics and strategies. Front Pharmacol. 2023;14:1231401. doi:10.3389/fphar.2023.1231401; 37593174 PMC10427877

[70] Goldberg RM, Sargent DJ, Morton RF, Fuchs CS, Ramanathan RK, Williamson SK, et al. A randomized controlled trial of fluorouracil plus leucovorin, irinotecan, and oxaliplatin combinations in patients with previously untreated metastatic colorectal cancer. J Clin Oncol. 2023;41(19):3461–8. doi:10.1200/JCO.22.02759; 37379691

[71] Kawai S, Takeshima N, Hayasaka Y, Notsu A, Yamazaki M, Kawabata T, et al. Comparison of irinotecan and oxaliplatin as the first-line therapies for metastatic colorectal cancer: a meta-analysis. BMC Cancer. 2021;21(1):116. doi:10.1186/s12885-021-07823-7; 33541293 PMC7863255

[72] Peipert JD, Roydhouse J, Tighiouart M, Henry NL, Kim S, Hays RD, et al. Overall side effect assessment of oxaliplatin toxicity in rectal cancer patients in NRG oncology/NSABP R04. Qual Life Res. 2024;33(11):3069–79. doi:10.1007/s11136-024-03746-5; 39080091 PMC11541265

[73] Aboeita NM, Fahmy SA, El-Sayed MMH, Azzazy HME, Shoeib T. Enhanced anticancer activity of nedaplatin loaded onto copper nanoparticles synthesized using red algae. Pharmaceutics. 2022;14(2):418. doi:10.3390/pharmaceutics14020418; 35214150 PMC8877422

[74] Chikazawa K, Netsu S, Imai K, Ishiguro A, Kimura A, Wang L, et al. Nedaplatin use in patients with hypersensitivity reaction episodes to carboplatin. Taiwan J Obstet Gynecol. 2020;59(4):546–50. doi:10.1016/j.tjog.2020.05.013; 32653127

[75] Yu Q, Lan T, Ma Z, Wang Z, Zhang C, Jiang Y, et al. Lobaplatin induces apoptosis in T24 and 5637 bladder cancer cells by regulating Bcl-2 and Bax expression and inhibiting the PI3K/Akt signaling pathway. Transl Androl Urol. 2023;12(8):1296–307. doi:10.21037/tau-23-376; 37680227 PMC10481196

[76] Tsvetkova D, Ivanova S. Application of approved cisplatin derivatives in combination therapy against different cancer diseases. Molecules. 2022;27(8):2466. doi:10.3390/molecules27082466; 35458666 PMC9031877

[77] Huang X, Zhou H, Jiao R, Liu H, Qin C, Xu L, et al. Supramolecular chemotherapy: host-guest complexes of Heptaplatin-Cucurbit[7]uril toward colorectal normal and tumor cells. Langmuir. 2021;37(18):5475–82. doi:10.1021/acs.langmuir.0c03603; 33913723

[78] Wang X, Wang M, Cai M, Shao R, Xia G, Zhao W. Miriplatin-loaded liposome, as a novel mitophagy inducer, suppresses pancreatic cancer proliferation through blocking POLG and TFAM-mediated mtDNA replication. Acta Pharm Sin B. 2023;13(11):4477–501. doi:10.1016/j.apsb.2023.07.009; 37969736 PMC10638513

[79] Zhang C, Kang T, Wang X, Song J, Zhang J, Li G. Stimuli-responsive platinum and ruthenium complexes for lung cancer therapy. Front Pharmacol. 2022;13:1035217. doi:10.3389/fphar.2022.1035217; 36324675 PMC9618881

[80] Lin K, Zhao ZZ, Bo HB, Hao XJ, Wang JQ. Applications of ruthenium complex in tumor diagnosis and therapy. Front Pharmacol. 2018;9:1323. doi:10.3389/fphar.2018.01323; 30510511 PMC6252376

[81] Riisom M, Eade L, Tremlett WDJ, Hartinger CG. The aqueous stability and interactions of organoruthenium compounds with serum proteins, cell culture medium, and human serum. Metallomics. 2022;14(7):mfac043. doi:10.1093/mtomcs/mfac043; 35751650 PMC9314723

[82] Deng Z, Gao P, Yu L, Ma B, You Y, Chan L, et al. Ruthenium complexes with phenylterpyridine derivatives target cell membrane and trigger death receptors-mediated apoptosis in cancer cells. Biomaterials. 2017;129:111–26. doi:10.1016/j.biomaterials.2017.03.017; 28340357

[83] Levina A, Chetcuti ARM, Lay PA. Controversial role of transferrin in the transport of ruthenium anticancer drugs. Biomolecules. 2022;12(9):1319. doi:10.3390/biom12091319; 36139158 PMC9496346

[84] Kanaoujiya R, Meenakshi, Srivastava S, Singh R, Mustafa G. Recent advances and application of ruthenium complexes in tumor malignancy. Mat Today: Proc. 2023;72(6):2822–7.

[85] Tang H, Guo X, Yu W, Gao J, Zhu X, Huang Z, et al. Ruthenium(II) complexes as mitochondrial inhibitors of topoisomerase induced A549 cell apoptosis. J Inorg Biochem. 2023;246:112295. doi:10.1016/j.jinorgbio.2023.112295; 37348172

[86] Althobaiti F, Sahyon HA, Shanab MMAH, Aldhahrani A, Helal MA, Khireldin A, et al. A comparative study of novel ruthenium(III) and iron(III) complexes containing uracil; docking and biological studies. J Inorg Biochem. 2023;247:112308. doi:10.1016/j.jinorgbio.2023.112308; 37441923

[87] Maikoo S, Xulu B, Mambanda A, Mkhwanazi N, Davison C, de la Mare JA, et al. Biomolecular interactions of cytotoxic ruthenium compounds with thiosemicarbazone or benzothiazole schiff base chelates. ChemMedChem. 2022;17(20):e202200444. doi:10.1002/cmdc.202200444; 36041073 PMC9826503

[88] Ceramella J, Troiano R, Iacopetta D, Mariconda A, Pellegrino M, Catalano A, et al. Synthesis of novel *N*-heterocyclic carbene-ruthenium (II) complexes, “precious” tools with antibacterial, anticancer and antioxidant properties. Antibiotics. 2023;12(4):693. doi:10.3390/antibiotics12040693; 37107055 PMC10135378

[89] Legina MS, Nogueira JJ, Kandioller W, Jakupec MA, González L, Keppler BK. Biological evaluation of novel thiomaltol-based organometallic complexes as topoisomerase IIα inhibitors. J Biol Inorg Chem. 2020;25(3):451–65. doi:10.1007/s00775-020-01775-2; 32193613 PMC7186247

[90] Ling YY, Li ZY, Mu X, Kong YJ, Hao L, Wang WJ, et al. Self-assembly of a ruthenium-based cGAS-STING photoactivator for carrier-free cancer immunotherapy. Eur J Med Chem. 2024;275:116638. doi:10.1016/j.ejmech.2024.116638; 38950489

[91] Del Pino JMV, Scalambra F, Bermejo-Casadesús C, Massaguer A, García-Maroto F, Romerosa A. Study of the biological activity of photoactive bipyridyl-Ru(II) complexes containing 1,3,5-triaza-7-phosphaadamantane (PTA). J Inorg Biochem. 2023;246:112291. doi:10.1016/j.jinorgbio.2023.112291; 37352655

[92] Ballester FJ, Hernández-García A, Santana MD, Bautista D, Ashoo P, Ortega-Forte E, et al. Photoactivatable ruthenium complexes containing minimal straining Benzothiazolyl-1,2,3-triazole chelators for cancer treatment. Inorg Chem. 2024;63(14):6202–16. doi:10.1021/acs.inorgchem.3c04432; 38385171 PMC11005040

[93] Gandioso A, Izquierdo-García E, Mesdom P, Arnoux P, Demeubayeva N, Burckel P, et al. Ru(II)-cyanine complexes as promising photodynamic photosensitizers for the treatment of hypoxic tumours with highly penetrating 770 nm near-infrared light. Chemistry. 2023;29(61):e202301742. doi:10.1002/chem.202301742; 37548580

[94] Santos LS, Silva VR, de Castro MVL, Dias RB, Valverde LF, Rocha CAG, et al. New ruthenium-xanthoxylin complex eliminates colorectal cancer stem cells by targeting the heat shock protein 90 chaperone. Cell Death Dis. 2023;14(12):832. doi:10.1038/s41419-023-06330-w; 38102125 PMC10724293

[95] Silva VR, Santos LS, de Castro MVL, Dias RB, Valverde LF, Rocha CAG, et al. A novel ruthenium complex with 5-fluorouracil suppresses colorectal cancer stem cells by inhibiting Akt/mTOR signaling. Cell Death Discov. 2023;9(1):460. doi:10.1038/s41420-023-01759-6; 38104089 PMC10725484

[96] Wang ZF, Huang XQ, Wu RC, Xiao Y, Zhang SH. Antitumor studies evaluation of triphenylphosphine ruthenium complexes with 5,7-dihalo-substituted-8-quinolinoline targeting mitophagy pathways. J Inorg Biochem. 2023;248:112361. doi:10.1016/j.jinorgbio.2023.112361; 37659141

[97] Ramírez-Rivera S, Pizarro S, Gallardo M, Gajardo F, Delgadillo A, De La Fuente-Ortega E, et al. Anticancer activity of two novel ruthenium compounds in gastric cancer cells. Life Sci. 2018;213:57–65. doi:10.1016/j.lfs.2018.10.024; 30326218

[98] Carrillo E, Ramírez-Rivera S, Bernal G, Aquea G, Tessini C, Thomet FA. Water-soluble Ru(II)-anethole compounds with promising cytotoxicity toward the human gastric cancer cell line AGS. Life Sci. 2019;217:193–201. doi:10.1016/j.lfs.2018.12.010; 30528721

[99] Liu T, Pan C, Shi H, Huang T, Huang YL, Deng YY, et al. Cytotoxic cis-ruthenium(III) bis(amidine) complexes. Dalton Trans. 2023;52(25):8540–8. doi:10.1039/D3DT00328K; 37000490

[100] Cao M, Fan B, Zhen T, Das A, Wang J. Ruthenium biochanin—a complex ameliorates lung carcinoma through the downregulation of the TGF-β/PPARγ/PI3K/TNF-α pathway in association with caspase-3-mediated apoptosis. Toxicol Res. 2023;39(3):455–75. doi:10.1007/s43188-023-00177-1; 37398567 PMC10313601

[101] Michlewska S, Wójkowska D, Watala C, Skiba E, Ortega P, de la Mata FJ, et al. Ruthenium metallodendrimer against triple-negative breast cancer in mice. Nanomed. 2023;53:102703. doi:10.1016/j.nano.2023.102703; 37591367

[102] Happl B, Brandt M, Balber T, Benčurová K, Talip Z, Voegele A, et al. Synthesis and preclinical evaluation of radiolabeled [103Ru] BOLD-100. Pharmaceutics. 2023;15(11):2626. doi:10.3390/pharmaceutics15112626; 38004604 PMC10674160

[103] Lu Y, Zhu D, Le Q, Wang Y, Wang W. Ruthenium-based antitumor drugs and delivery systems from monotherapy to combination therapy. Nanoscale. 2022;14(44):16339–75. doi:10.1039/D2NR02994D; 36341705

[104] Machado PHA, Paixão DA, Lino RC, de Souza TR, de Souza Bontempo NJ, Sousa LM, et al. A selective CuII complex with 4-fluorophenoxyacetic acid hydrazide and phenanthroline displays DNA-cleaving and pro-apoptotic properties in cancer cells. Sci Rep. 2021;11(1):24450. doi:10.1038/s41598-021-03909-1; 34961767 PMC8712526

[105] Ji P, Wang P, Chen H, Xu Y, Ge J, Tian Z, et al. Potential of copper and copper compounds for anticancer applications. Pharmaceuticals. 2023;16(2):234. doi:10.3390/ph16020234; 37259382 PMC9960329

[106] Lelièvre P, Sancey L, Coll JL, Deniaud A, Busser B. The multifaceted roles of copper in cancer: a trace metal element with dysregulated metabolism, but also a target or a bullet for therapy. Cancers. 2020;12(12):3594. doi:10.3390/cancers12123594; 33271772 PMC7760327

[107] Khan AQ, Rashid K, AlAmodi AA, Agha MV, Akhtar S, Hakeem I, et al. Reactive oxygen species (ROS) in cancer pathogenesis and therapy: an update on the role of ROS in anticancer action of benzophenanthridine alkaloids. Biomed Pharmacother. 2021;143:112142. doi:10.1016/j.biopha.2021.112142; 34536761

[108] Nakamura H, Takada K. Reactive oxygen species in cancer: current findings and future directions. Cancer Sci. 2021;112(10):3945–52. doi:10.1111/cas.v112.10. 34286881 PMC8486193

[109] Gao W, Huang Z, Duan J, Nice EC, Lin J, Huang C. Elesclomol induces copper-dependent ferroptosis in colorectal cancer cells via degradation of ATP7A. Mol Oncol. 2021;15(12):3527–44. doi:10.1002/mol2.v15.12. 34390123 PMC8637554

[110] Ghasemi P, Shafiee G, Ziamajidi N, Abbasalipourkabir R. Copper nanoparticles induce apoptosis and oxidative stress in SW480 human colon cancer cell line. Biol Trace Elem Res. 2023;201(8):3746–54. doi:10.1007/s12011-022-03458-2; 36274109

[111] Al-Zharani M, Qurtam AA, Daoush WM, Eisa MH, Aljarba NH, Alkahtani S, et al. Antitumor effect of copper nanoparticles on human breast and colon malignancies. Environ Sci Pollut Res. 2021;28(2):1587–95. doi:10.1007/s11356-020-09843-5; 32851522

[112] Masuri S, Vaňhara P, Cabiddu MG, Moráň L, Havel J, Cadoni E, et al. Copper(II) phenanthroline-based complexes as potential anticancer drugs: a walkthrough on the mechanisms of action. Molecules. 2022;27(1):49. 10.3390/molecules27010049 PMC8746828 35011273

[113] Xi YH, Yan X, Bigdeli F, Zhang Q, Esrafili L, Hanifehpour Y, et al. Two new Cu (II) complexes based on 5-fluorouracil-1-yl acetic acid and N-donor ligands: investigation of their interaction with DNA and anticancer activity. Appl Organomet Chem. 2022;36(1):e6458. doi:10.1002/aoc.v36.1.

[114] Emami F, Aliomrani M, Tangestaninejad S, Kazemian H, Moradi M, Rostami M. Copper-curcumin-bipyridine dicarboxylate complexes as anticancer candidates. Chem Biodivers. 2022;19(10):e202200202. doi:10.1002/cbdv.v19.10. 36163613

[115] Machado JF, Marques F, Pinheiro T, Villa de Brito MJ, Scalese G, Pérez-Díaz L, et al. Copper(I)-thiosemicarbazone complexes with dual anticancer and antiparasitic activity. ChemMedChem. 2023;18(14):e202300074. doi:10.1002/cmdc.v18.14. 37098105

[116] Garufi A, Scarpelli F, Ricciardi L, Aiello I, D’Orazi G, Crispini A. New copper-based metallodrugs with anti-invasive capacity. Biomolecules. 2023;13(10):1489. doi:10.3390/biom13101489; 37892171 PMC10604694

[117] Moreno-Alcántar G, Picchetti P, Casini A. Gold complexes in anticancer therapy: from new design principles to particle-based delivery systems. Angew Chem Int Ed Engl. 2023;62(22):202218000. doi:10.1002/anie.v62.22. 36847211

[118] Sánchez Delgado GY, Arvellos JFA, Paschoal DFS, Dos Santos HF. Role of the enzymatic environment in the reactivity of the AuIII-C^N^C anticancer complexes. Inorg Chem. 2021;60(5):3181–95. doi:10.1021/acs.inorgchem.0c03521; 33600154

[119] Khan HA, Al-Hoshani A, Isab AA, Alhomida AS. A Gold(III) complex with potential anticancer properties. ChemistrySelect. 2022;7(45):e202202956. doi:10.1002/slct.v7.45.

[120] Yue S, Luo M, Liu H, Wei S. Recent advances of gold compounds in anticancer immunity. Front Chem. 2020;8:543. doi:10.3389/fchem.2020.00543; 32695747 PMC7338717

[121] Zhang J, Li Y, Fang R, Wei W, Wang Y, Jin J, et al. Organometallic gold(I) and gold(III) complexes for lung cancer treatment. Front Pharmacol. 2022;13:979951. doi:10.3389/fphar.2022.979951; 36176441 PMC9513137

[122] Yeo CI, Ooi KK, Tiekink ERT. Gold-based medicine: a paradigm shift in anti-cancer therapy? Molecules. 2018;23(6):1410. doi:10.3390/molecules23061410; 29891764 PMC6100309

[123] Quero J, Ruighi F, Osada J, Gimeno MC, Cerrada E, Rodriguez-Yoldi MJ. Gold(I) complexes bearing alkylated 1,3,5-triaza-7-phosphaadamantane ligands as thermoresponsive anticancer agents in human colon cells. Biomedicines. 2021;9(12):1848. doi:10.3390/biomedicines9121848; 34944664 PMC8698759

[124] Gil-Moles M, Concepción Gimeno M. The therapeutic potential in cancer of terpyridine-based metal complexes featuring group 11 elements. ChemMedChem. 2024;19(10):e202300645. doi:10.1002/cmdc.v19.10. 38328860

[125] Gukathasan S, Obisesan OA, Saryazdi S, Ratliff L, Parkin S, Grossman RB, et al. A conformationally restricted gold(III) complex elicits antiproliferative activity in cancer cells. Inorg Chem. 2023;62(32):13118–29. doi:10.1021/acs.inorgchem.3c02066; 37530672 PMC11268950

[126] Sousa SA, Leitão JH, Silva RAL, Belo D, Santos IC, Guerreiro JF, et al. On the path to gold: monoanionic Au bisdithiolate complexes with antimicrobial and antitumor activities. J Inorg Biochem. 2020;202:110904. doi:10.1016/j.jinorgbio.2019.110904; 31671298

[127] Augello G, Azzolina A, Rossi F, Prencipe F, Mangiatordi GF, Saviano M, et al. New insights into the behavior of NHC-gold complexes in cancer cells. Pharmaceutics. 2023;15(2):466. doi:10.3390/pharmaceutics15020466; 36839788 PMC9963827

[128] Kim JH, Ofori S, Parkin S, Vekaria H, Sullivan PG, Awuah SG. Anticancer gold(III)-bisphosphine complex alters the mitochondrial electron transport chain to induce: *in vivo* tumor inhibition. Chem Sci. 2021;12(21):7467–79. doi:10.1039/D1SC01418H; 34163837 PMC8171344

[129] Jiang J, Cao B, Chen Y, Luo H, Xue J, Xiong X, et al. Alkylgold(III) complexes undergo unprecedented photo-induced β-hydride elimination and reduction for targeted cancer therapy. Angew Chem Int Ed Engl. 2022;61(16):e202201103. doi:10.1002/anie.v61.16. 35165986

[130] Giuso V, Yang J, Forté J, Dossmann H, Daniel C, Gourlaouen C, et al. Binuclear Biphenyl Organogold(III) complexes: synthesis, photophysical and theoretical investigation, and anticancer activity. Chempluschem. 2023;88(11):e202300303. doi:10.1002/cplu.v88.11. 37610058

[131] Fahmy HM, Mosleh AM, El-Sayed AA, El-Sherif AA. Novel palladium(II) and Zinc(II) schiff base complexes: synthesis, biophysical studies, and anticancer activity investigation. J Trace Elem Med Biol. 2023;79:127236. doi:10.1016/j.jtemb.2023.127236; 37285632

[132] Seddik RG, Shoukry AA, Rashidi FB, Salah-Eldin DS. Investigation on CT-DNA and protein interaction of new Pd(II) complexes involving ceftazidime and 3-Amino-1,2,3-triazole: synthesis, characterization, biological impact, anticancer evaluation, and molecular docking approaches. Chem Biodivers. 2023;20(12):e202301170. doi:10.1002/cbdv.v20.12. 37850505

[133] Feizi-Dehnayebi M, Dehghanian E, Mansouri-Torshizi H. Biological activity of bis-(morpholineacetato) palladium(II) complex: preparation, structural elucidation, cytotoxicity, DNA-/serum albumin-interaction, density functional theory, in-silico prediction and molecular modeling. Spectrochim Acta A Mol Biomol Spectrosc. 2022;281:121543. doi:10.1016/j.saa.2022.121543; 35797947

[134] Jovičić Milić SS, Jevtić VV, Radisavljević SR, Petrović BV, Radojević ID, Raković IR, et al. Synthesis, characterization, DNA interactions and biological activity of new palladium(II) complexes with some derivatives of 2-aminothiazoles. J Inorg Biochem. 2022;233:111857. doi:10.1016/j.jinorgbio.2022.111857; 35597043

[135] Liu Y, Li J, Chen M, Chen X, Zheng N. Palladium-based nanomaterials for cancer imaging and therapy. Theranostics. 2020;10(22):10057–74. doi:10.7150/thno.45990; 32929334 PMC7481408

[136] Hosseini-Kharat M, Rahimi R, Alizadeh AM, Zargarian D, Khalighfard S, Mangin LP, et al. Cytotoxicity, anti-tumor effects and structure-activity relationships of nickel and palladium S,C,S pincer complexes against double and triple-positive and triple-negative breast cancer (TNBC) cells. Bioorg Med Chem Lett. 2021;43:128107. doi:10.1016/j.bmcl.2021.128107; 33991624

[137] Taskinen I, Jääskeläinen S, Koshevoy I, Hirva P. Palladium and platinum complexes of primary 2-pyridinethioamide. Solid State Sci. 2023;146:107374. doi:10.1016/j.solidstatesciences.2023.107374.

[138] Hosseini MJ, Jafarian I, Farahani S, Khodadadi R, Tagavi SH, Naserzadeh P, et al. New mechanistic approach of inorganic palladium toxicity: impairment in mitochondrial electron transfer. Metallomics. 2016;8(2):252–9. doi:10.1039/C5MT00249D; 26739318

[139] Nadeem S, Bolte M, Ahmad S, Fazeelat T, Tirmizi SA, Rauf MK, et al. Synthesis, crystal structures and, antibacterial and antiproliferative activities *in vitro* of palladium(II) complexes of triphenylphosphine and thioamides. Inorganica Chimica Acta. 2010;363(13):3261–9. doi:10.1016/j.ica.2010.06.015.

[140] Zhou XQ, Wang P, Ramu V, Zhang L, Jiang S, Li X, et al. *In vivo* metallophilic self-assembly of a light-activated anticancer drug. Nat Chem. 2023;15(7):980–7. doi:10.1038/s41557-023-01199-w; 37169984 PMC10322715

[141] Ouyang R, Wang S, Feng K, Liu C, Silva DZ, Chen Y, et al. Potent saccharinate-containing palladium(II) complexes for sensitization to cancer therapy. J Inorg Biochem. 2023;244:112205. doi:10.1016/j.jinorgbio.2023.112205; 37028114

[142] Krstic A, Pavic A, Avdovic E, Markovic Z, Stevanovic M, Petrovic I. Coumarin-Palladium(II) complex acts as a potent and non-toxic anticancer agent against pancreatic carcinoma cells. Molecules. 2022;27(7):2115. doi:10.3390/molecules27072115; 35408514 PMC9000835

[143] Schoofs H, Schmit J, Rink L. Zinc toxicity: understanding the limits. Molecules. 2024;29(13):3130. doi:10.3390/molecules29133130; 38999082 PMC11243279

[144] Bilensoy E, Varan C. Is there a niche for zinc oxide nanoparticles in future drug discovery? Expert Opin Drug Discov. 2023;18(9):943–5. doi:10.1080/17460441.2023.2230152; 37381770

[145] Qu Z, Liu Q, Kong X, Wang X, Wang Z, Wang J, et al. A systematic study on zinc-related metabolism in breast cancer. Nutrients. 2023;15(7):1703. doi:10.3390/nu15071703; 37049543 PMC10096741

[146] Wiesmann N, Tremel W, Brieger J. Zinc oxide nanoparticles for therapeutic purposes in cancer medicine. J Mater Chem B. 2020;8(23):4973–89. doi:10.1039/D0TB00739K; 32427264

[147] Liang JY, Liu YY, Zou J, Franklin RB, Costello LC, Feng P. Inhibitory effect of zinc on human prostatic carcinoma cell growth. Prostate. 1999;40(3):200–7. doi:10.1002/(ISSN)1097-0045. 10398282 PMC4465333

[148] Li D, Stovall DB, Wang W, Sui G. Advances of zinc signaling studies in prostate cancer. Int J Mol Sci. 2020;21(2):667. doi:10.3390/ijms21020667; 31963946 PMC7014440

[149] Zhang P, Li Y, Tang X, Guo R, Li J, Chen Y, et al. Zinc enhances chemosensitivity to paclitaxel in PC-3 prostate cancer cells. Oncol Rep. 2018;40:2269–77. doi:10.3892/or.2018.6622; 30106439

[150] Xue YN, Yu BB, Liu YN, Guo R, Li JL, Zhang LC, et al. Zinc promotes prostate cancer cell chemosensitivity to paclitaxel by inhibiting epithelial-mesenchymal transition and inducing apoptosis. Prostate. 2019;79(6):647–56. doi:10.1002/pros.23772; 30714183

[151] George BP, Rajendran NK, Houreld NN, Abrahamse H. Rubus capped zinc oxide nanoparticles induce apoptosis in MCF-7 breast cancer cells. Molecules. 2022;27(20):6862. doi:10.3390/molecules27206862; 36296460 PMC9611499

[152] Mohamed SY, Elshoky HA, El-Sayed NM, Fahmy HM, Ali MA. Ameliorative effect of zinc oxide-chitosan conjugates on the anticancer activity of cisplatin: approach for breast cancer treatment. Int J Biol Macromol. 2024;257:128597. doi:10.1016/j.ijbiomac.2023.128597; 38056740

[153] Alallam B, Doolaanea AA, Alfatama M, Lim V. Phytofabrication and characterisation of zinc oxide nanoparticles using pure curcumin. Pharmaceuticals. 2023;16(2):269. doi:10.3390/ph16020269; 37259414 PMC9960272

[154] Li G, Chen J, Xie Y, Yang Y, Niu Y, Chen X, et al. White light increases anticancer effectiveness of iridium(III) complexes toward lung cancer A549 cells. J Inorg Biochem. 2024;259:112652. doi:10.1016/j.jinorgbio.2024.112652; 38945112

[155] Lv A, Li G, Zhang P, Tao R, Li X, Ren X, et al. Design and anticancer behaviour of cationic/neutral half-sandwich iridium (III) imidazole-phenanthroline/phenanthrene complexes. J Inorg Biochem. 2024;257:112612. doi:10.1016/j.jinorgbio.2024.112612; 38761579

[156] Kasparkova J, Novohradsky V, Ruiz J, Brabec V. Photoactivatable, mitochondria targeting dppz iridium (III) complex selectively interacts and damages mitochondrial DNA in cancer cells. Chem Biol Interact. 2024;392:110921. doi:10.1016/j.cbi.2024.110921; 38382705

[157] Hu H, Zhang F, Sheng Z, Tian S, Li G, Tang S, et al. Synthesis and mitochondria-localized iridium (III) complexes induce cell death through pyroptosis and ferroptosis pathways. Eur J Med Chem. 2024;268:116295. doi:10.1016/j.ejmech.2024.116295; 38437750

[158] Chen L, Tang H, Chen W, Wang J, Zhang S, Gao J, et al. Mitochondria-targeted cyclometalated iridium (III) complexes: dual induction of A549 cells apoptosis and autophagy. J Inorg Biochem. 2023;249:112397. doi:10.1016/j.jinorgbio.2023.112397; 37844533

[159] Yang C, Deng X, Tang Y, Tang H, Xia C. Natural products reverse cisplatin resistance in the hypoxic tumor microenvironment. Cancer Lett. 2024;598:217116. doi:10.1016/j.canlet.2024.217116; 39002694

[160] Cheng Y, Fan Y, Zhao Y, Huang D, Li X, Zhang P, et al. Tislelizumab plus platinum and etoposide versus placebo plus platinum and etoposide as first-line treatment for extensive-stage SCLC (RATIONALE-312): a multicenter, double-blind, placebo-controlled, randomized, phase 3 clinical trial. J Thorac Oncol. 2024;19(7):1073–85. doi:10.1016/j.jtho.2024.03.008; 38460751

[161] Reck M, Barlesi F, Yang JC, Westeel V, Felip E, Özgüroğlu M, et al. Avelumab versus platinum-based doublet chemotherapy as first-line treatment for patients with high-expression programmed death-Ligand 1-positive metastatic NSCLC: primary analysis from the phase 3 JAVELIN Lung 100 trial. J Thorac Oncol. 2024;19(2):297–313. doi:10.1016/j.jtho.2023.09.1445; 37748693

[162] Mok T, Nakagawa K, Park K, Ohe Y, Girard N, Kim HR, et al. Nivolumab plus chemotherapy in epidermal growth factor receptor-mutated metastatic non-small-cell lung cancer after disease progression on epidermal growth factor receptor tyrosine kinase inhibitors: final results of CheckMate 722. J Clin Oncol. 2024;42(11):1252–64. doi:10.1200/JCO.23.01017; 38252907 PMC11095864

[163] Chen M, Zhang H, Cui WX, Chen MY, Cheng XP. The effect of selenium yeast in the prevention of adverse reactions related to platinum-based combination therapy in patients with malignant tumors. Eur Rev Med Pharmacol Sci. 2023;27(21):10499–506; 37975373 10.26355/eurrev_202311_34326

[164] Koeneman BJ, Schreibelt G, Gorris MAJ, Hins-de Bree S, Westdorp H, Ottevanger PB, et al. Dendritic cell vaccination combined with carboplatin/paclitaxel for metastatic endometrial cancer patients: results of a phase I/II trial. Front Immunol. 2024;15:1368103. doi:10.3389/fimmu.2024.1368103; 38444861 PMC10912556

[165] Khanmammadov NJ, Doğan İ., Okay NS, Azizy A, Saip P, Aydiner A. Comparative analysis of doublet chemotherapy regimens plus bevacizumab in patients with recurrent ovarian cancer. Medicine. 2024;103(1):e36750. doi:10.1097/MD.0000000000036750; 38181291 PMC10766248

[166] Leijen S, Burgers SA, Baas P, Pluim D, Tibben M, Van Werkhoven E, et al. Phase I/II study with ruthenium compound NAMI-A and gemcitabine in patients with non-small cell lung cancer after first line therapy. Invest New Drugs. 2015;33(1):201–14. doi:10.1007/s10637-014-0179-1; 25344453

[167] Lentz F, Drescher A, Lindauer A, Henke M, Hilger RA, Hartinger CG, et al. Pharmacokinetics of a novel anticancer ruthenium complex (KP1019, FFC14A) in a phase I dose-escalation study. Anticancer Drugs. 2009;20(2):97–103. doi:10.1097/CAD.0b013e328322fbc5; 19209025

[168] Kulkarni GS, Lilge L, Nesbitt M, Dumoulin-White RJ, Mandel A, Jewett MAS. A phase 1b clinical study of intravesical photodynamic therapy in patients with Bacillus Calmette-Guérin-unresponsive non-muscle-invasive bladder cancer. Eur Urol Open Sci. 2022;41:105–11. doi:10.1016/j.euros.2022.04.015; 35813250 PMC9257636

